# Estimating Dispersal Kernels for Stream Periphyton Using Drifting Lake Algae

**DOI:** 10.1002/ece3.74334

**Published:** 2026-09-13

**Authors:** Daniel Zamorano, Claudio I. Meier, Tilman M. Davies, Travis Ingram, Ursula Romero, Christoph D. Matthaei

**Affiliations:** ^1^ Department of Zoology University of Otago Dunedin New Zealand; ^2^ Instituto de Ciencias Marinas y Limnológicas, Facultad de Ciencias Universidad Austral de Chile Valdivia Chile; ^3^ Department of Civil Engineering University of Memphis Memphis Tennessee USA; ^4^ Department of Mathematics & Statistics University of Otago Dunedin New Zealand; ^5^ Laboratorio de Limnología, Facultad de Ciencias Universidad de Chile Santiago Chile

**Keywords:** long‐distance dispersal process, metacommunity, phytoplankton, settling experiment, stream algae

## Abstract

Dispersal patterns are a key component of many ecological and evolutionary processes, yet they remain understudied in microbial species due to technical challenges. Stream periphyton is a microbial group of public interest and is frequently used as a bioindicator. While high dispersal capability along streams has been suggested for periphyton, no previous studies have empirically estimated dispersal distances for these taxa. Methods for dispersal quantification cannot be used for periphyton species within the river continuum because it is challenging to identify the source point of a given microalga. We propose therefore using phytoplankton drifting from a lake outlet as a proxy for dispersing stream periphyton. The aims of this study were, first, to test the pertinence of using phytoplankton as a dispersal proxy for periphyton, and second, to estimate dispersal distances of stream periphyton. We conducted settling velocity experiments to estimate and compare the buoyancy of phytoplankton and periphyton taxa, confirming that phytoplankton were broadly suitable as proxies. In a lake outlet stream near Dunedin, New Zealand, we exposed ceramic tiles as standardized substrates at different distances from the outlet to collect settling microalgae. A statistical hydraulic model was developed to describe the potential dispersal distances obtained by settling velocities. Dispersal distances were estimated for seven taxa using densities from tiles and for 26 taxa using settling velocities and the statistical hydraulic model. Median travel distances were around 500 m downstream of the lake outlet, with phytoplankton rarely detected further than 2 km from the source. Overall, our findings pointed to stream turbulence and morphological variability within the same algal taxa as the main determinants of dispersal distance. Our novel approach demonstrates how phytoplankton can be used to infer dispersal distances for stream periphyton, representing an important milestone in quantitatively characterizing dispersal patterns in stream periphyton communities.

## Graphical abstract

Summary of methods and procedures organized by aim and specific objectives. Drifting lake algae used as a proxy for periphyton dispersal.

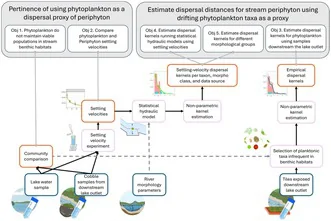

## 1 Introduction

How far species can disperse is one of the oldest questions in ecology, due to its importance in disentangling the spatial dynamics of populations and communities (Darwin and Wallace 1858; Briggs 2009). Information on species' dispersal distances and frequency can be applied to a wide range of research questions, conservation planning, and invasive species management programs. For example, understanding the distance and frequency of dispersal events is crucial for assessing metapopulation stability, interpreting patterns of genetic variation, estimating the recolonization probability of extinct patches, and evaluating the establishment probability of new subpopulations (Trakhtenbrot et al. 2005; Baguette et al. 2013). For threatened species, dispersal information is necessary to precisely estimate extinction risk and the effectiveness of management actions, which in turn improves the design and prioritization of management strategies (Driscoll et al. 2014). On the other hand, it has been observed that invasive species capable of combining short‐ and long‐distance dispersal exhibit faster invasion velocity by facilitating low‐density invasion fronts, thereby avoiding density‐dependent constraints (Marco et al. 2011). In this context, the study of dispersal and movement ecology has become one of the fastest‐growing fields in the past decade (Nathan et al. 2022).

Despite recent advances, microbial dispersal processes remain understudied. A first understanding of microbial biogeography was reached less than two decades ago (Martiny et al. 2006; Jenkins et al. 2007), and even now we are unsure whether macroecological rules drive microbial processes (Dickey et al. 2021). One of the microbial groups of primary public interest due to its role in freshwater biomonitoring worldwide is stream periphyton (Zhao et al. 2023), whose dispersal processes are still unclear. Periphyton is the matrix‐enclosed community growing attached to submerged surfaces, composed of microalgae, cyanobacteria, bacteria, fungi, and archaea (Battin et al. 2016). For simplicity, the term “periphyton algae” is used to describe this community from here onwards. Periphyton algae are the base of most autochthonous production in stream food webs (Ishikawa et al. 2014) and can provide important ecosystem services such as water purification (Rooney et al. 2020) and carbon sequestration (Yan et al. 2021).

The existing literature about periphyton dispersal indicates high population connectivity within catchments, among catchments, and among larger geographical regions (Soininen et al. 2016; Pajunen et al. 2017; Medeiros et al. 2020; Oliveira et al. 2023; Fuß et al. 2025), implying high dispersal capabilities. This evidence is supported by the fact that periphyton algae have been detected in the air (Sahu and Tangutur 2015), on birds (Szabó et al. 2022), and on mammals (Leone et al. 2014). Combined with the algae's capability to resist droughts (Colls et al. 2021) or to produce spores or akinetes (Cirés et al. 2013), these observations support the idea of high capability for dispersal beyond the geographical boundaries.

Despite high connectivity among catchments, flow‐mediated dispersal along streams remains the principal migration pathway for stream periphyton (Chaput‐Bardy et al. 2009; Dong et al. 2016). Currently, high connectivity within catchments is deduced from analyses of species presence‐absence matrices (Medeiros et al. 2020, 2022; Fuß et al. 2025). There are no estimates of dispersal distances for periphyton along streams, which represents a considerable gap in the literature (Jenkins et al. 2007). At the same time, while many periphyton species are sessile and live attached to wet substrates, other periphyton species have a degree of mobility at the scale of μm to cm, such as gliding diatoms or flagellated algae (Lauterborn 1896; Cohn 2001; Poulsen et al. 2022). The relationships between periphyton motile traits at the microscale and dispersal processes along streams have not been explored yet (Custer et al. 2022). Estimating periphyton species dispersal distance is vital because it allows moving on from the long‐standing idea of “everything everywhere, but the environment selects” (as proposed by Baas‐Becking 1934) to potentially establish specific dispersal ranges for periphyton populations according to the species' functional traits and landscape configuration.

A wide variety of methodologies have been developed for quantifying dispersal patterns. For example, for plant seeds researchers often employ propagule traps exposed at varying distances from a source point to measure propagule travel distances (Dupont et al. 2006; Herrmann et al. 2016). Other methodologies focus on tracking individual trajectories using radio tracking, GPS tags, or mark‐and‐recapture (Revilla and Wiegand 2008; Altobelli et al. 2022; Payne et al. 2022). Further approaches involve mechanistic or statistical modeling of passively dispersed propagules (Robledo‐Arnuncio et al. 2004; Arritt et al. 2007).

Despite the broad range of existing methodologies for estimating dispersal distances, none of them are suitable for stream periphyton species. Periphyton dispersal patterns could potentially be assessed via propagule traps below a “source point.” However, identifying such a source point for multiple algal species within a river continuum is extremely challenging due to strong environmental gradients at small spatial scales (Erős and Schmera 2010; Domisch et al. 2015). While downstream dispersal distances of particulate organic matter have been estimated using distinctive particles, many of these are unlikely to be good proxies for living algae, such as radiolabeled organic material (Monaghan et al. 2001; Ishikawa et al. 2012), fluorescent spores (Wanner and Pusch 2000), or lake sediment (Hünken and Mutz 2007). Nevertheless, some studies have used lake phytoplankton to estimate dispersal distances (Maciolek and Tunzi 1968; Ock and Takemon 2010), which is very promising in the context of our study. By using lake phytoplankton drifting down a lake outlet stream to estimate stream periphyton dispersal ranges, a clear source point (the lake outlet) is established within the river continuum, and this approach allows for the estimation of taxon‐specific dispersal patterns. However, a key question in the context of our study is whether lake phytoplankton taxa can serve as a valid proxy for the dispersal of stream periphyton species.

Phytoplankton is the photosynthetic community living in the surface layers of standing waters (Harris 2012). Phytoplankton occupies a different ecological niche than periphyton, which is attached to stream or lake beds and wet surfaces in general. Nevertheless, at the taxonomic level, phytoplankton is composed of the same clades as periphyton (e.g., green algae, diatoms, and cyanobacteria), and there are many genera whose species exhibit both benthic and planktonic habits (e.g., *Cladophora* (Dodds and Gudder 1992), *Anabaena* (Katrianna et al. 2008), or *Gomphonema* (Abarca et al. 2020)). Similarly, in a study on motility traits, buoyancy and flagellated motility were present in both periphyton and phytoplankton taxa (Raven and Beardall 2022). Thus, while periphyton and phytoplankton are ecologically different, they share many similarities at the taxonomic and functional levels. Consequently, we argue that using phytoplankton as a proxy for periphyton dispersal processes is a suitable starting point for the challenging task of estimating periphyton dispersal distances.

First, we aimed to evaluate the suitability of phytoplankton as a dispersal proxy for periphyton in a lake outlet stream. For this aim, we had two specific objectives:

1. To confirm that phytoplankton taxa do not maintain viable populations in stream benthic habitats, thus establishing that phytoplankton organisms observed in our study stream were drifting from a source point (the lake outlet). We carried out statistical analyses to compare the presence and density of phytoplankton taxa in lake water and stream cobble samples. Moreover, we reviewed the literature and selected planktonic taxa that have not been described in benthic habitats for our further analyses.
2. To compare the settling velocities of phytoplankton and periphyton taxa. If the settling velocities of both taxa are similar, we can assume that the drift patterns observed for phytoplankton will be similar to periphyton drift patterns. We estimated settling velocities using settling velocity experiments, which have been widely used in freshwater or marine studies (Kamykowski et al. 1992; Kearns and Hunter 2001; Aumack and Juhl 2015; Bouma‐Gregson et al. 2017; Durante et al. 2019) to infer the vertical movements of phytoplankton.

Following from this first step, our second aim was to estimate dispersal distances for stream periphyton taxa using drifting phytoplankton taxa as a dispersal proxy, assessing the dispersal patterns observed according to their morphological traits and environmental conditions. In this context, we established three additional specific objectives:

3 To estimate dispersal kernels using phytoplankton empirical data. Dispersal kernels are defined as probability density functions describing the distribution of the post‐dispersal locations of a dispersing organism relative to its source point (Nathan et al. 2012; Rogers et al. 2019). We assessed the abundances of several phytoplankton taxa downstream of a lake outlet, using phytoplankton densities as inputs for kernel densities.
4 To estimate dispersal kernels for phytoplankton and periphyton taxa using settling velocities calculated from settling velocity experiments, and using a statistical hydraulic model to simulate dispersal. We developed such a model for our study stream and, using the estimated settling velocities for a number of taxa, simulated virtual dispersal trips and calculated dispersal kernels from the settling velocities. While settling velocities could be used as a dispersal proxy, we consider that given the context of stream turbulent flow, they need to be applied to a simulation/model that properly considers them in the context of dispersal. Thus, this model allowed us to directly compare potential dispersal events between periphyton and phytoplankton taxa.
5 To estimate settling velocity‐based dispersal kernels for taxa grouped by morphology, allowing the assessment of dispersal patterns based on functional traits. Settling velocities were estimated per morphological group, and this information was then applied to the statistical hydraulic model used to simulate dispersal.

**FIGURE 1:**
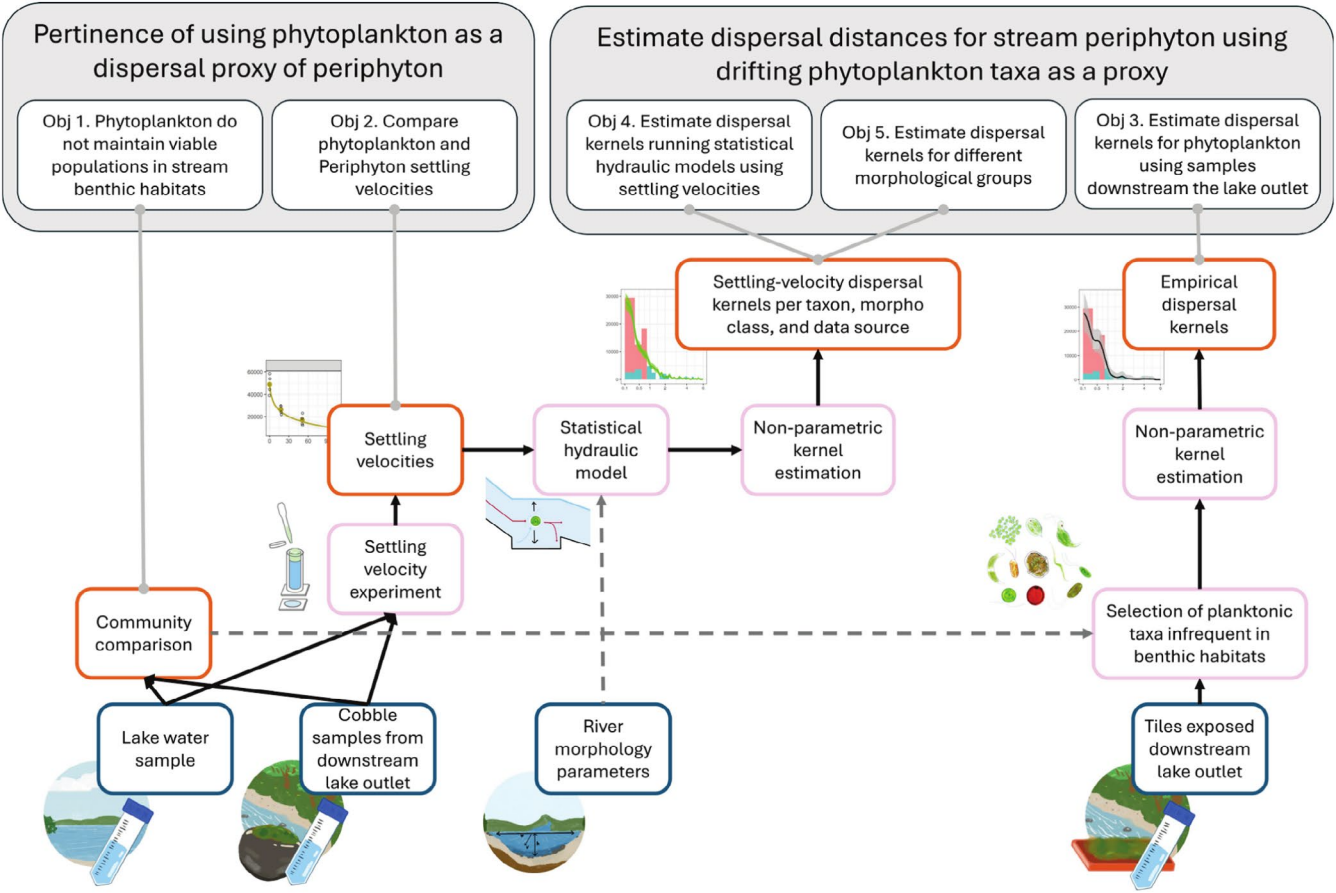
Summary of methods and procedures organized by aim and specific objectives. At the top of the diagram, the main aims and specific objectives are connected by lines to the main outputs to which they relate, which are connected to the main procedures used for their estimation. Boxes in orange represent the main outputs, boxes in pink represent procedures such as laboratory or statistical experiments, and boxes in blue represent information or samples from the field. Solid black lines represent the main inputs to procedures, and dashed gray lines represent secondary inputs. Please note that the objectives are not in numerical order to improve the diagram layout.

Given the complexity of our study, the objectives, methods, and procedures are summarized in Figure 1.

## 2 Methods and Procedures

### 2.1 Study Area

**FIGURE 2:**
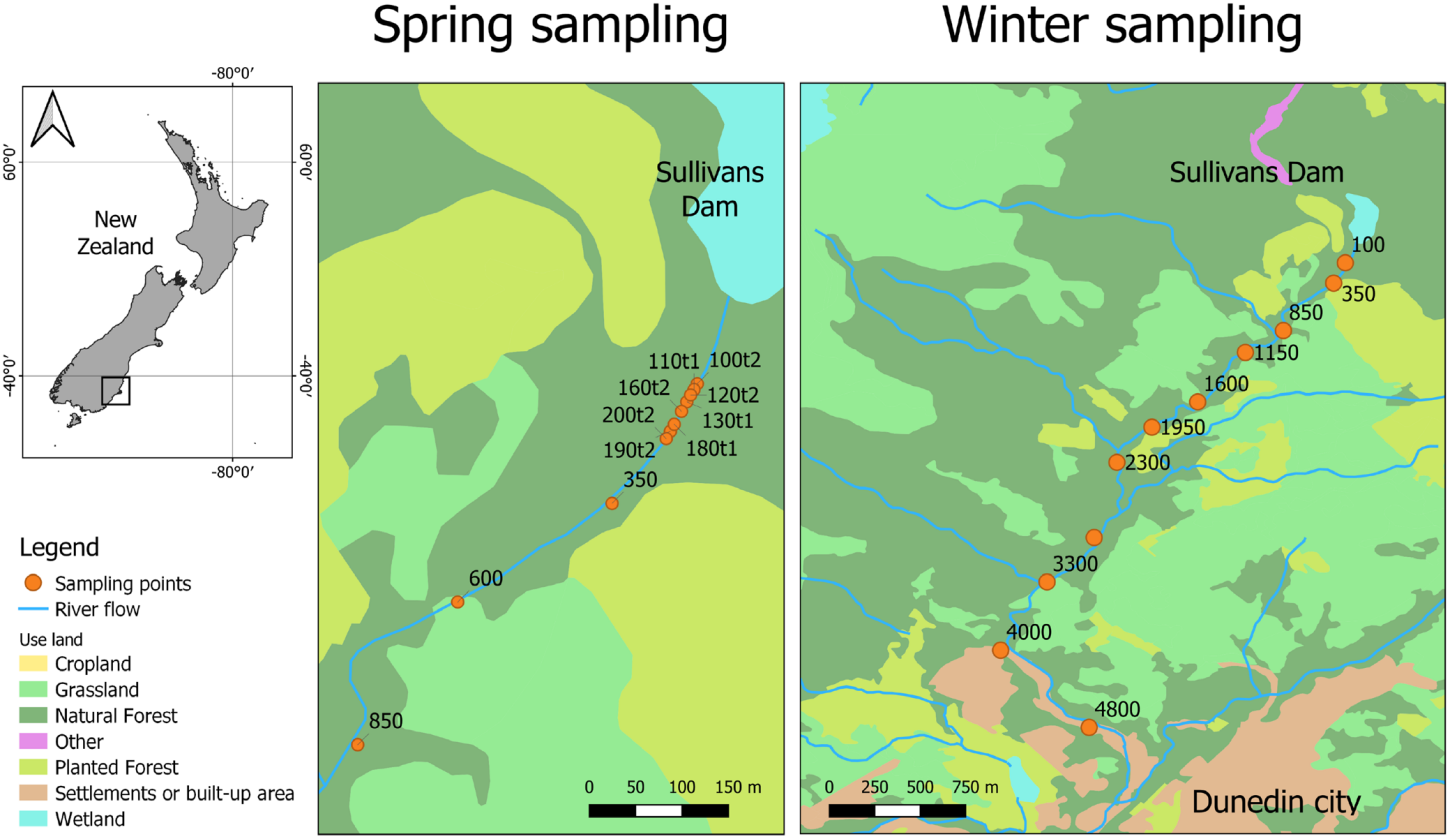
Map of the study area near the city of Dunedin, New Zealand. The orange points show the locations of the tiles exposed during the spring and winter sampling periods in the Water of Leith. For the spring sampling, the suffixes indicate whether tiles were exposed during the first two weeks (t1) or the second two weeks (t2) of this period. Despite the proximity to Dunedin, the studied reaches of the Leith are mostly surrounded by native forest.

Our study was conducted in third‐ and fourth‐order reaches of a stream, the Water of Leith, below a reservoir, Sullivans Dam, near the city of Dunedin in the South Island of New Zealand (Figure 2). The lake has a surface outlet with only a 1‐m drop below the dam. Although a few tributaries enter the Leith between 1 km below the dam and further downstream, these are mostly small (first or second order), and none are connected to another lentic body of water. The upper half of the Leith catchment (above the reservoir) is mostly covered by native forest, whereas the lower half flows through suburban and urban areas of Dunedin (Otago Regional Council 2008).

### 2.2 Study Design and Sampling Methods

Samples of phytoplankton from lake water (hereafter, lake samples) and samples of periphyton from submerged cobbles downstream of the lake outlet (hereafter, cobble samples) were collected to fulfill three aims: to analyze community composition and determine the presence of phytoplankton taxa in benthic habitats (Objective 1); to be included as organic material for settling velocity experiments (Objective 2); and to select planktonic taxa with low prevalence in benthic habitats to estimate empirical dispersal kernels (Objective 3) (Figure 1). Lake samples were composed of 1‐L samples of surface water collected during two sampling periods (see below). Cobble samples were collected from submerged cobbles at four sites along the Leith: at 350 and 850 m below the lake outlet during spring, and at 1950 and 7700 m during winter. At each site, we collected five surface cobbles (diameter 10–20 cm) located at least 2 m from each other. These cobble sampling locations were chosen to characterize the periphyton community of the sites where we exposed tiles as standardized substrates (see below).

To obtain densities of drifting phytoplankton in the stream to estimate empirical dispersal kernels (Objective 3), unglazed ceramic tiles (10 × 10 × 1 cm) as standardized substrates were exposed for 14 days in the stream at different distances downstream from the lake outlet (hereafter, tile samples). The first sampling period was in October–November 2020 (Austral spring). We exposed two tiles at each of eight sites (100, 110, 120, 130, 160, 180, 190, and 200 m downstream), and four tiles each at three sites (350, 600, and 850 m downstream) (Figure 2a). Tiles placed between 0 m (the dam wall) and 100 m downstream were washed away because this short section is channelized with a concrete bed and therefore suffers from high bed shear stress. Exploratory analyses showed unclear dispersal trends for phytoplankton densities when we sampled to 850 m downstream; therefore, we decided to extend the sampling distance in a second campaign. This second sampling period was conducted in June 2021 (Austral winter). In this period, we exposed four tiles at 10 sites (100, 350, 850, 1150, 1600, 1950, 2300, 3300, 4000, and 4800 m downstream) (Figure 2b). This sampling design resulted in 27 tiles in spring (28 minus one that was washed away at site 190 m) and 33 tiles in winter (40 minus seven washed away, one each at sites 100 m, 350 m, 1150 m, 1950 m, 2300 m, 3300 m, and 4000 m). All collected tiles, lake, and cobbles samples were immediately labeled in the field, placed into individual zip‐lock bags on ice in the dark, and transported to the laboratory on the same day.

To characterize microhabitat conditions where tiles were exposed, we measured water depth (range 5.5–47 cm, mean 19.5 cm) and flow velocity (measured 1 cm above tiles, range 0.01–0.53 cm·s^−1^, mean 0.15 cm·s^−1^) at the location of each tile. Depth was measured with a ruler, and flow velocity with an electromagnetic flow meter (FlowMate 2000; Marsh‐McBirney Inc., Frederick, MD, USA). Microhabitat depth and flow velocity were used to calibrate the statistical hydraulic model (Objectives 4 and 5) to explore the role of habitat morphology in the settling probability of drifting algae, and to assess any possible bias given the tile location in the channel.

### 2.3 Laboratory Procedures

For each cobble sample, we removed 2 × 2 cm area of periphyton from its upper surface using a scalpel and a toothbrush, rinsing the resulting slurry into a Falcon tube. For each tile, all periphyton on the top surface was removed with a toothbrush into a tray and the resulting slurry was rinsed into Falcon tubes (volume 50 mL). The sample was then preserved by adding Lugol's iodine. Due to the presence of fine sediment on tiles and cobbles from the spring sampling period, these samples were sonicated using a bath sonicator to separate algal cells from the fine sediment (3 min at 40 kHz; Transsonic T 1040/H, Elma GmbH & Co KG, Singen, Germany). Each cobble and lake sample was split into two parts. One part was preserved with Lugol's iodine for community composition analysis, and the second part was stored in a refridgerator (4°C) for up to 3 days for use in the settling experiment (see below).

When community composition was assessed for lake, cobble, and tile samples, we enumerated at least 200 algal or cyanobacterial cells per sample by examining 15–50 microscope fields at 400× magnification under an inverted microscope (Zeiss Axiovert 25, Jena, Germany). Taxa were identified to genus using standard keys (Foged 1979; Entwisle et al. 1997; Biggs and Kilroy 2000; Moore 2000; Bellinger and Sigee 2010; Spaulding et al. 2021; Jüttner et al. 2023). Filamentous or colonial taxa were treated as “natural counting units” by counting as one unit each filament or algal colony found in a sample (Charles et al. 2002), and these units are referred to as “cells” henceforth.

### 2.4 Settling Velocity Experiments

These experiments were carried out in Thalheim settling columns, which are acrylic columns commonly used to settle algae from water samples. We used three different column sizes: small (height 18 mm—volume 10 mL), medium (51 mm—25 mL), and large (106 mm—50 mL). Each column was first filled with distilled water, and we then added at the top an algal sample of 2.5 mL with a pipette. After 5 min, we removed the chamber from the bottom and analyzed the sample under the microscope (see Figure S1 and the legend for details). For the lake samples, we repeated the experiment four times per column size using winter samples. Regrettably, the trials for the lake spring samples failed, and we could not include them in this analysis. For cobble samples, we repeated the experiment nine times per column size, using five cobble samples from the site at 350 m and four cobble samples from the site at 850 m. Before the settling experiments, we analyzed a subsample of each lake or cobble samples (hereafter “reference samples”) under the microscope to determine how many cells were available to settle. Cobble samples were diluted to match the lake sample cell densities, thus reaching similar settling densities between experiments and allowing us to perform more replicate trials with cobble samples.

**FIGURE 3:**
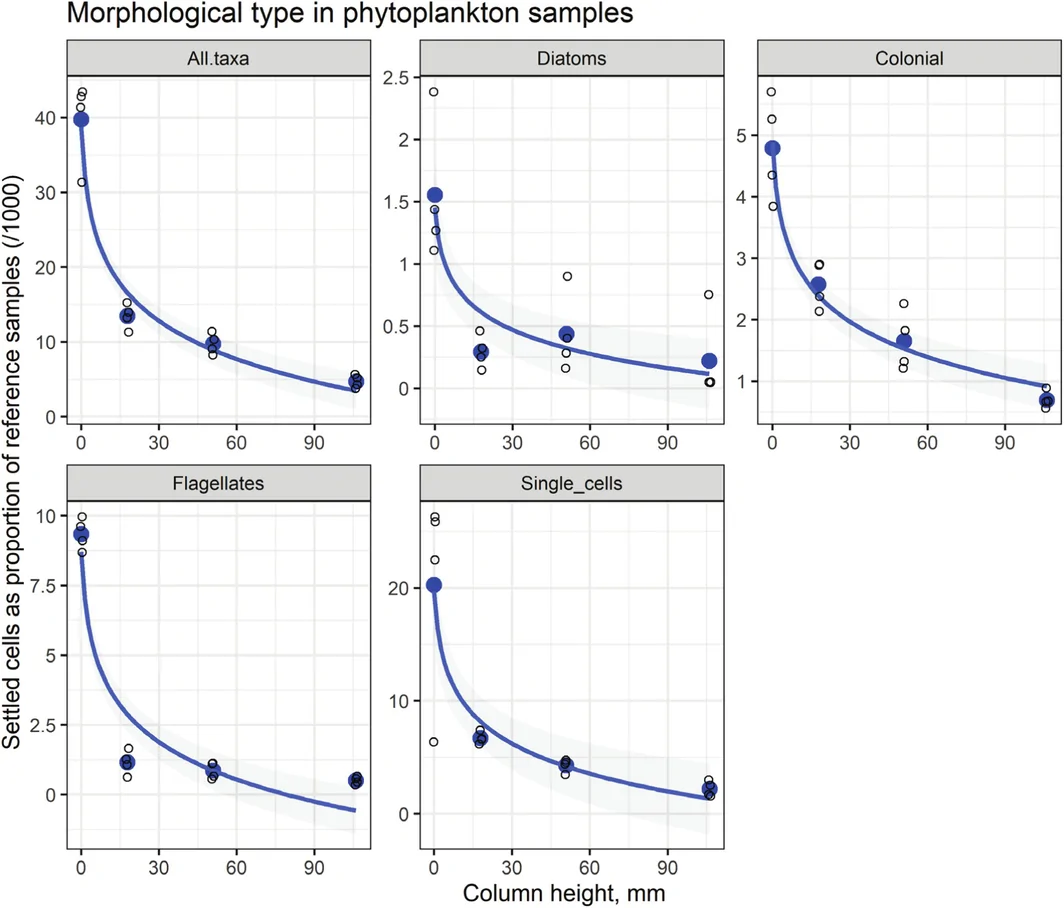
Example of settling velocity curve fitting for a subset of our data. Settled cell proportions according to column height are shown for total cell density and for phytoplankton taxa clustered by morphological classes. Empty dots represent column replicate density values, and solid dots represent means per column height. Curves were fitted using a log‐normal distribution.

All settling velocity data computation, further statistical hydraulic modeling and statistical analyses were carried out in R (R Core Team 2023). All algal densities recorded in each settling trial were divided by the density recorded in the corresponding reference sample, and 1 was then subtracted from this proportion. With these data, we estimated a cumulative distribution function by fitting a log‐normal function, where the settled cell proportion was the response variable and the settling velocity (column height/5 min) was the predictor variable. After applying a selection criterion based on data availability, we ended up successfully fitting settling velocity functions for 26 algal taxa (see Figure S2 for more details about the distribution‐fitting process and taxon selection). We used these log‐normal distribution functions to calculate the median, first and third quartiles, and 10th and 90th percentiles of each settling velocity. Taxa were grouped into six taxonomic/morphological classes (adapted from Biggs and Kilroy 2000) as follows: cyanobacteria, colonial or multi‐celled (henceforth called colonial), single‐celled (single‐cell), filamentous (including filamentous cyanobacteria), flagellated, and diatoms. An example of the log‐normal curve fitting is given in Figure 3, where cell density is plotted against the column height of each experiment for different morphological classes.

### 2.5 Statistical Hydraulic Model

The primary challenge in our modeling was to simulate a river reach incorporating flow turbulence, in a way that would allow contrasting the opposite effects of vertical turbulent fluctuations against those of the algal settling velocity. Developing a 3D hydrodynamic model would have been excessively complex, and would lack any generality, while a steady‐state, uniform flow model would have been overly simplistic. We therefore developed a 1‐D statistical hydraulic model of a virtual river reach, composed of pool‐riffle sequences, where each pool and riffle was in turn made up of a discrete number of habitats. Individual algal cells were assumed to be transported from one habitat to the next. For each of these habitats, we estimated the Rouse number (*R*_*o*_) as the ratio of the algal settling velocity (*V*_*s*_) to the intensity of the upward vertical turbulent fluctuations, calculated as the product of the von Kármán constant (*κ*) and the shear velocity (u*).

(1) $$R_{o}=\frac{V_{s}}{\kappa \cdot u^{*}}$$

The Rouse number (*R*_*o*_) typically assesses suspended sediment concentration profiles and sediment transport in a flowing, turbulent fluid.

For a known stream discharge, the shear velocity (u*) in each habitat was calculated based on the geometry of the reach (width, length, roughness of each riffle and pool, plus the longitudinal slope for riffles). Each habitat was assigned a uniform depth, width, and roughness. Riffles had a specified slope, with depth determined through Gauckler‐Manning equation, while pools had no slope. Values of the morphological parameters were assigned randomly to each habitat from normal distributions adapted to represent the sampled river. Further model details are provided in Appendix S1.

To convert *R*_*o*_ into a settling probability, we applied the following logistic function (Equation 2), where the settling probability is ~0 when *R*_*o*_ < 0.8, 0.5 when *R*_*o*_ = 1.65, and ~1 when *R*_*o*_ > 2.5. This function was initially fitted following a load transport mode and then adjusted to yield dispersal distances comparable to the empirical dispersal kernels.

(2) $$P\left(R_{o}\right)=\frac{1}{1+e^{-4.5\;\left(R_{o}-1.65\right)}}$$

Each simulated habitat was sectioned into thirds of its width, and the settling probability as a function of *R*_*o*_ was calculated in each section. Algal settlement or the continuation of drifting was then determined by random sampling weighted by the settling probability. This process was repeated sequentially, with the algae encountering new pool‐riffle sequences downstream until settling occurred. For each taxon and morphological class, we ran 1000 simulations, each using settling velocities from the log‐normal distribution obtained from settling experiments. This resulted in 1000 dispersal distances per taxon and morphological class, which we used to construct settling‐velocity dispersal kernels.

### 2.6 Taxa Selection for Empirical Dispersal Kernels

To assess the dispersal patterns recorded by our tiles, we selected only plankton taxa whose reproductive possibility within the stream periphyton mats was close to zero. Hence, we determined the 20 most abundant phytoplankton taxa in our lake water samples and reviewed the ecological literature on these. We reviewed several books and journal articles with autecological descriptions of these taxa (Biggs and Kilroy 2000; Reynolds et al. 2002; Bellinger and Sigee 2010; Burgos Romero 2010; Sant'Anna et al. 2012; González and Inostroza 2017), as well as descriptions in the AlgaeBase database (Guiry and Guiry 2021), and we specifically looked for studies that reported the presence of these taxa in stream periphyton mats. The resulting final selection of eight taxa used to assess algal dispersal kernels were: *Closterium* spp., *Euglena* spp., *Dictyosphaerium* spp., *Peridinium* spp., *Cryptomonas* spp., *Phacus* spp., and *Chlamydomonas* spp. To obtain a dispersal kernel that summarized all available data, we also included a variable with the combined density of all selected taxa (variable name “all taxa”), thus resulting in eight empirical dispersal kernels in total.

We implemented a set of analyses to determine how habitat morphology might influence algal settling on tiles exposed in the stream. We used generalized linear mixed models (GLMM) to evaluate the effects of water depth and flow velocity on the density of the seven selected taxa for kernel analysis and the total selected taxa phytoplankton density. Also, total microalgae density was evaluated using a GLMM to compare patterns between the selected phytoplankton taxa and the benthic community. These models included season and distance from the lake outlet as random factors to account for the non‐independence of samples collected during the two seasons and at the different locations along the stream. For the eight selected taxa, we added a zero‐inflation component to account for the high number of zeros in the datasets. Response variables and predictors were normalized using the square root, log_10_, and the function *bestNormalize::bestNormalize* (Peterson 2018). We used the function *glmmTMB::glmmTMB* for the GLMM (Brooks et al. 2017), and model residuals were assessed using the function *DHARMa::testResiduals* (Hartig 2022).

### 2.7 Dispersal Kernel Estimation

Exploratory data analysis indicated that estimating dispersal kernels with well‐known parametric distributions (e.g., as in Nathan et al. 2012) would be suboptimal due to abruptly observed changes in algae densities. Therefore, we applied kernel density estimation (KDE), a nonparametric smoothing method for estimating probability distributions (see e.g., Wand and Jones 1994). While highly flexible, the implementation of KDE involves a trade‐off in smoothing: greater smoothing produces a more biased estimate, whereas less smoothing increases variability and noise. To achieve an “optimal” level of smoothing, we used a data‐driven bandwidth selection technique (biased cross‐validation). Confidence intervals for each dispersal kernel were estimated via bootstrapping, generating 100 resampled kernels to calculate the 0.05 and 0.95 quantiles at each distance point. KDEs were obtained using the function *evmix::dbckden* (Hu and Scarrott 2018). A comprehensive description of KDE and the choices made in our implementation thereof is given in Appendix S2.

We estimated KDE‐based dispersal kernels using three datasets: the selected phytoplankton dataset from tiles downstream of the lake outlet (Objective 3, empirical dispersal kernels), and the dispersal distances datasets generated with the statistical hydraulic model using the settling velocities of periphyton taxa, phytoplankton taxa (Objective 4), and morphological classes (Objective 5) obtained from cobble and lake samples. We refer to the dispersal kernels derived from model simulations as settling‐velocity dispersal kernels, if the settling velocity was estimated using cobble samples, we refer to them as settling periphyton dispersal kernels; if using lake samples, we refer to them as settling phytoplankton dispersal kernels. For the empirical dispersal kernels, we carried out a jittering process for the tile cell density dataset where, by defining a series of intervals/zones between tiles, we uniformly jittered a proportionate number of observations prior to implementing kernel estimation (Appendix S2). Finally, to characterize dispersal distances of each dispersal kernel, quantiles 0.1, 0.25, 0.5, 0.75, and 0.9 were estimated using the function *evmix::qbckden* (Hu and Scarrott 2018).

## 3 Results

### 3.1 Phytoplankton Presence in Benthic Habitat

The mean relative abundance of the eight selected taxa was consistently 60% higher in the lake samples than in the cobble samples. The only exception was *Closterium* spp., which showed a 50% higher relative abundance in the cobble periphyton. *Peridinium* spp., *Euglena* spp., and *Rhodomonas* spp. were consistently ranked among the five most common taxa in the lake water samples (Table S1).

Microhabitat conditions did not affect the densities of the selected phytoplankton taxa collected on the tiles. Zero‐inflation models showed no significant results for depth and flow velocity as predictors of the selected taxon densities or total cell density in either the counts or zero‐inflation components (Table S2). The only exception was a significant but weak negative correlation between *Chlamydomonas* spp. counts and flow velocity (*r* = −0.37). Due to the lack of significant results, the impact of microhabitat conditions on our periphyton data was not further explored.

### 3.2 Settling Experiment

For the cobble samples, 29 of the 71 recorded taxa contained enough data to attempt to fit a lognormal distribution. Of those taxa, 21 (72%) showed a significant fit with a lognormal distribution (Table S3). For the lake samples, 15 of 36 taxa had enough data, and eight (53%) showed a significant fit with a lognormal distribution. All taxonomic/morphological algal classes contained enough data to fit lognormal distributions to both types of samples. However, for cyanobacteria forms, no model fit significantly, whereas for filamentous forms, only the river data fit a significant model.

For the taxa in the lake samples, we detected an average settling velocity of 0.049 mm·s^−1^ (interquartile range 0.011–0.201 mm·s^−1^). For the taxa in the cobble samples, the average settling velocity was 0.035 (0.008–0.117) mm·s^−1^ (Table S1). Considering the cobble and lake samples, the average settling velocity of the selected phytoplankton taxa for the empirical dispersal kernel was 0.051 (0.019–0.14) mm·s^−1^. By comparison, the average settling velocity of the remaining taxa, which were considered to be mainly periphytic, was 0.084 (0.034–0.25) mm·s^−1^. Consequently, while the phytoplankton settling velocity was slower on average, the interquartile ranges of the respective settling velocities observed for phytoplankton and periphyton taxa overlapped. Thus, we believe it is reasonable to compare the potential travel distances for plankton and periphyton taxa. While recognizing that periphytic taxa recorded slightly faster settling velocities overall.

### 3.3 Dispersal Patterns

**FIGURE 4:**
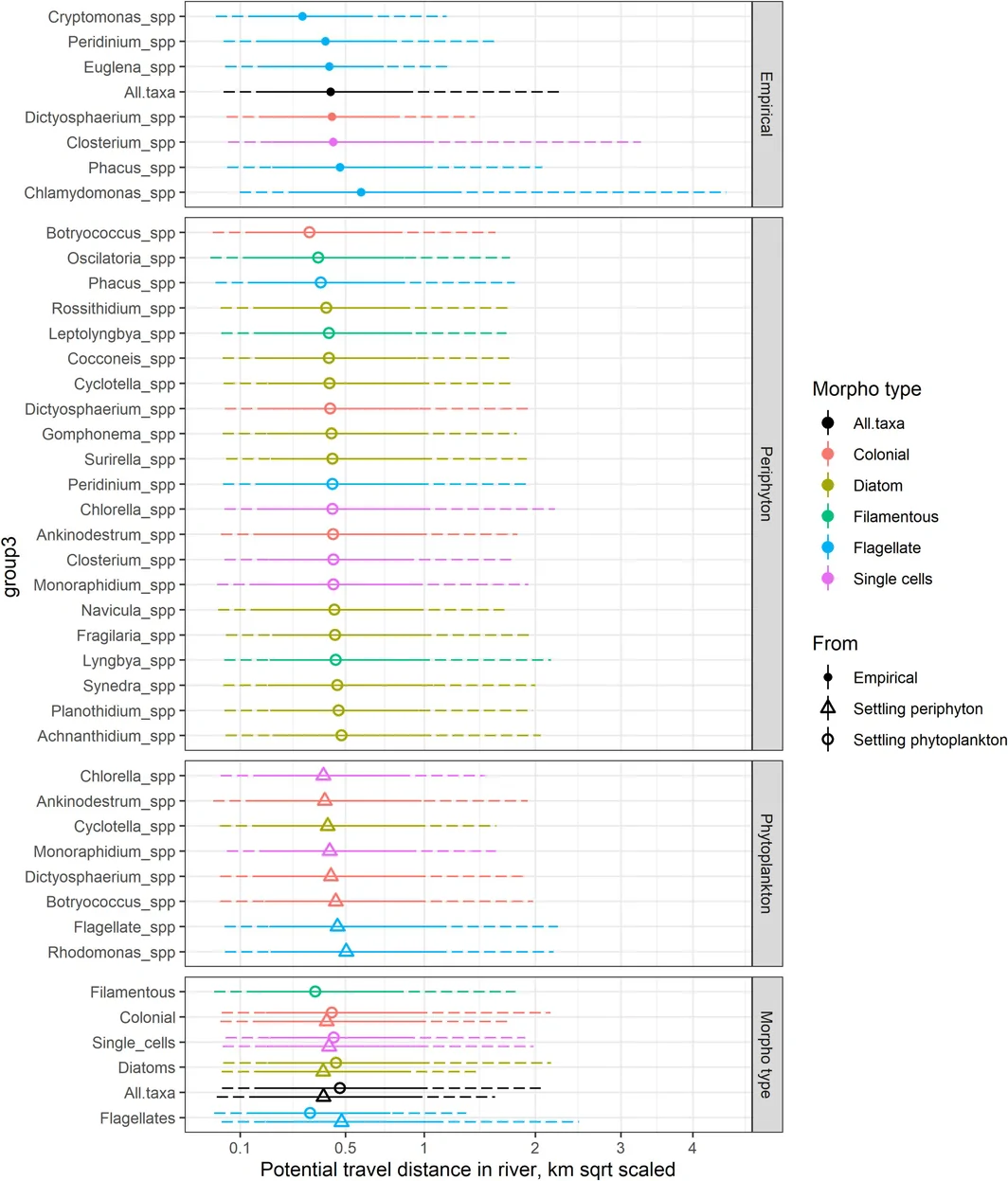
Potential travel distances in the Water of Leith for total algal cell density, each of the five taxonomic/morphological algal classes, and the 26 individual algal taxa. For each response variable, the dot at the center of the line represents the median travel distance, the continuous part of the line shows the interquartile range of dispersal distances, and the dashed parts of the line represent the range between the 10th and 90th percentiles. Response variables are ordered by increasing median dispersal distance within each of the two categories shown. Travel distances were obtained from settling velocity dispersal kernels from river cobble samples (Periphyton), settling velocity dispersal kernels from lake water samples (Phytoplankton), and empirical dispersal kernels from exposed tiles in the river (Kernel).

In general, the dispersal distances estimated using the three datasets overlapped. For the empirical dispersal kernels, we calculated an average dispersal distance of 433 m (interquartile range 182–890 m). For the cobble dispersal kernels, the average distance was 434 (123–1070) m. Finally, for the settling phytoplankton dispersal kernels, the average distance was 418 (134–1127) m. Potential travel distances for all taxa were generally similar in the cobble and lake samples, both ranging approximately from 50 m to 2 km (Figure 4). Only two cases (the flagellates morphological group and *Botryococcus* spp.) exhibited notable differences between the cobble and lake samples, with both taxa showing longer travel distances for settling velocities from the lake samples. Potential travel distances for all taxa are shown in Figure 4, and further details about these results are provided in Table S3 for settling‐velocity dispersal kernels, and in Table S4 for the empirical kernels.

Regarding the travel distances calculated from the settling‐velocity dispersal kernels, the shortest median distance observed (329 m) was for *Botryococcus* spp. (a colonial form, from the river cobble samples), which had a median settling velocity of 0.0138 mm·s^−1^. Conversely, the longest median distance observed (505 m) was for the flagellated *Rhodomonas* spp. (from the lake samples), which had a median settling velocity of 0.00095 mm·s^−1^. Thus, a settling velocity 14 times faster (0.0138 vs. 0.00095 mm·s^−1^) corresponded to a median travel distance that was only about 200 m shorter (329 vs. 500 m). Regarding morphological groups (Figure 4), flagellated taxa showed both the shortest (330 m) and longest median distances (480 m) for the cobble and lake samples, respectively. Paralleling the results observed for settling velocities, diatoms, single cells, and flagellated exhibited longer median dispersal distances than colonial and filamentous taxa. However, the 90th percentile distance for colonial taxa (from lake water samples) exceeded 2 km, surpassed only by diatoms and flagellated taxa.

Regarding the empirical dispersal kernels estimated using data from the exposed tiles (Figure 4), the shortest median distance recorded was 299 m for the flagellated *Cryptomonas* spp., whereas the longest median distance was 586 m for the flagellated *Chlamydomonas* spp. – a difference of approximately 280 m. This range is slightly larger than the minimum‐maximum range observed for the settling‐velocity median distances (329–505 m). Across all taxa and dispersal kernel methods, the 10th percentile distance was consistently ~50 m, but the 90th percentile showed a broader range. In 10 of 37 cases, taxa exhibited distances over 2 km in the 90th percentile for the empirical kernels, with *Closterium* spp. reaching 3.2 km and *Chlamydomonas* spp. 4.5 km.

**FIGURE 5:**
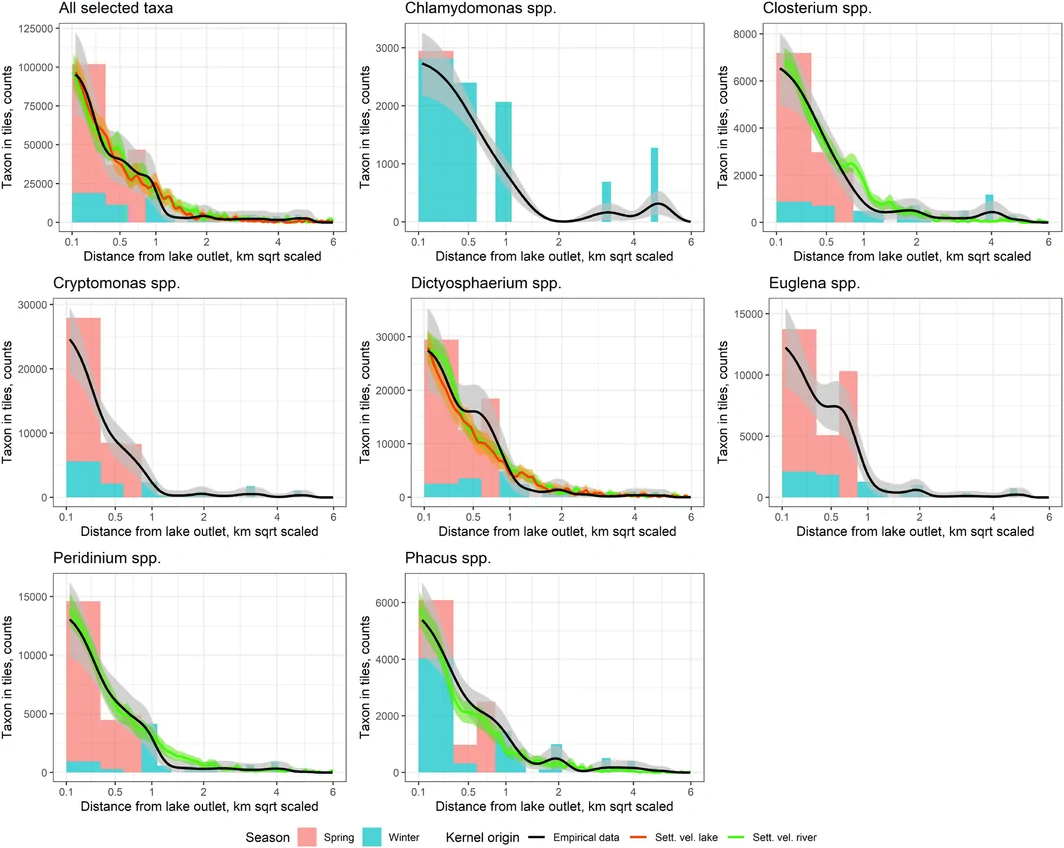
Cell density of the eight selected phytoplankton taxa and the combined cell density of these taxa in relation to the distance from the lake outlet. The color of each bar represents the sampling season (S = spring, W = winter). Curves with solid black lines represent the kernel distributions based on field data. Curves with dashed lines represent settling velocity dispersal kernels (green = river cobble samples, red = lake water samples). All dispersal kernels are shown with confidence intervals. Kernel distributions were scaled to match the axis values of each plot. For some taxa, it was impossible to estimate one or both of their theoretical kernel distributions due to a lack of data (see Methods for details).

The densities of the seven selected phytoplankton taxa and the combined density of all selected taxa on the tiles exposed in the Leith generally decreased with distance from the lake outlet, with density peaks occurring near the outlet (Figure 5). Overall, the empirical dispersal kernels effectively described the density patterns recorded on the tiles without overfitting. For example, in *Chlamydomonas* and *Closterium*, density peaks beyond 2 km were accurately captured by the kernels. When comparing kernel trajectories, the empirical dispersal kernels decreased almost linearly up to 1 km, with a minor second abundance peak occurring before 1 km for some taxa, then followed by relatively flat curves beyond 1 km (Figure 5). By contrast, the settling‐velocity dispersal kernels mostly showed a steady exponential decrease, with only minor variability due to density modes from the simulations. All three dispersal kernels could be estimated in two of the eight cases shown in Figure 5, for “All selected taxa” and *Dictyosphaerium* spp., and in both cases the three kernels overlapped.

## 4 Discussion

While the approach of using settling velocities to describe dispersal patterns in stream ecosystems has been previously employed in fine particulate organic matter studies (Cushing et al. 1993; Thomas et al. 2001; Newbold et al. 2005), our study includes two key novel aspects: (1) assessing dispersal processes for stream periphyton from a community perspective, and (2) incorporating two novel methodologies to determine both virtual and empirical dispersal distances. In general, we consider our method to be successful. The dispersal distances obtained from the statistical hydraulic simulations were similar to the distances observed from the empirical dispersal kernels. This indicates that settling velocity can be used to estimate travel distances and further allows us to extrapolate properly from the dispersal patterns of phytoplankton carried downstream to what is expected for periphyton in flow‐mediated dispersal. Interestingly, while the average planktonic settling velocity was slower, our estimated dispersal distance indicated similar ranges of values for periphytic and planktonic taxa, which highlights the role of turbulence in driving algal transportation.

### 4.1 Settling Velocities and Dispersal Distances

When compared to a literature review of settling velocities for phytoplankton (Durante et al. 2019), our interquartile range for all taxa (0.0008–0.021 mm·s^−1^) fit within the lower range of values in that review (0.00016–0.24 mm·s^−1^), suggesting relatively slow settling velocities in our study. In general, the taxa from the cobble and lake samples performed similarly during the settling experiments and shared the same ranges of settling velocities. Settling velocity depends first on cell density and second on particle size and shape (Durante et al. 2019), with shape being more relevant for high‐density particles than for low‐density particles (Dietrich 1982; Padisák et al. 2003). In our study, the morphology of the taxa selected as phytoplanktonic was spherical in most cases (except for *Closterium* spp. and *Dictyosphaerium* spp.), which could explain why the settling velocity values obtained for these taxa fit within the range of taxa with more diverse shape as, such as the periphyton taxa.

In general, our settling velocities for the taxonomic/morphological groups corroborate the relevant literature, with algal taxa with less complex shapes, such as single‐cell forms and diatoms, exhibiting slower settling velocities and therefore longer dispersal distances (by ~450 m) compared to filamentous forms or colonies (by ~300 m) (Padisák et al. 2003; Durante et al. 2019). However, for the flagellates, individuals from the river cobble and lake water samples showed very different settling velocities, with lake flagellates being much slower to settle. Most phytoplankton algae can control their buoyancy by actively modifying vacuole gas density, a mechanism that allows them to remain in benign environmental conditions (Bienfang et al. 1982; Choi et al. 2006; Reynolds 2006). While the impact of this settling‐control mechanism on our results was mitigated by providing homogeneous experimental conditions, flagellates can also swim actively instead of sinking when seeking resources (Reynolds 2006). Differences in this swimming behavior between river and lake flagellates could explain the pattern observed.

The considerable differences in settling velocities observed among the studied periphyton taxa did not result in substantial differences in median dispersal distances. If our experiment had been conducted in a system with laminar flow, the dispersal distances would perfectly mimic the distribution of settling velocities, given that the distance traveled downstream would be determined by the time required for the algae to settle. Instead, by adding the probabilistic role of flow turbulence to our experiment, we successfully replicated the patterns observed from the empirical data, which resulted in dispersal distances being homogenized among taxa. This finding emphasizes that periphyton dispersal processes in running waters are significantly affected by water turbulence, which adds a strong stochastic component to this dispersal. Consequently, in our study differences between algal taxa played a minor role in determining dispersal distances compared to habitat conditions and variation within the same taxon, which were the primary factors modulating settling probability. While the largest difference in median dispersal distance between taxa was approximately 200 m, the difference between the 10th and 90th percentiles for the same taxon was around 2 km. This result suggests that differences within the same taxon, likely driven by morphological variations, contributed more to dispersal variability than differences among taxa. As discussed above, because the environmental requirements of taxa may diverge, some of them may settle faster to avoid unfavorable conditions (Reynolds 2006), resulting in different settling velocities. Moreover, different environmental contexts can also determine biomass production, affecting microalgal cellular density (Okcu 2012; Yudiati et al. 2022). If settling velocity depends on cell condition, an unfavorable environmental context could result in a lower cell density, increasing the potential dispersal distance and thus favoring migration. Based on our findings, this mechanism could be the main source of dispersal variability within the same algal taxon, resulting in travel distances up to 20 times longer for certain individuals of the same taxa. Further research should be conducted to test this hypothesis.

### 4.2 Dispersal Processes for Stream Periphyton

The observed patterns of phytoplankton density on our tiles at different distances below the lake outlet suggest that the algal dispersal process includes a strong stochastic component. The pool‐riffle habitat sequence in our study stream, a sequence common in many streams and rivers (Montgomery and Buffington 1997), probably resulted in a series of different hydraulic conditions that, interacting with algal traits, created a high variability in algal settlement rates at the microhabitat scale. By segmenting our virtual river into pools and riffles and incorporating high turbulence in riffles and low turbulence in pools, we successfully replicated the results observed in the empirical dispersal kernels. Thus, we propose that river morphology and its interaction with the turbulent flow are the primary drivers of dispersal events, followed by differences in morphology and behavioral patterns within the same algal taxon, and finally, differences among algal taxa. Studies on fine particulate organic matter dispersal have provided expected travel distances of ~3 km for shallow and ~20 km for deep streams (Maciolek and Tunzi 1968; Ock and Takemon 2010), supporting the idea that algal dispersal patterns are highly habitat‐dependent. While further studies are necessary to apply our proposed hydraulic model to different river morphologies, we hypothesize that similar results will be observed in any river composed of riffles and pools, where turbulent flow will be the main driver of dispersal. However, it is more difficult to extrapolate our results to deep and/or slow‐flowing rivers, where the interaction between flow velocity and periphyton functional traits may result in unexpected dispersal patterns.

Although peaks of phytoplankton density disappeared below 1 km downstream of the lake outlet in our study, phytoplankton density never dropped to zero further downstream. This could be a consequence of river turbulence keeping the algae suspended for longer distances, and/or the capability of phytoplankton taxa to survive in the riverbed. For example, *Closterium* spp. has been described as highly periphytic by some authors (Biggs and Kilroy 2000), in contrast with the fairly long dispersal distances indicated by our kernel models. Our study cannot determine with certainty the phytoplankton sources for each sampled tile, as they could be from single dispersal events or a consequence of phytoplankton establishing viable populations in the riverbed (as might be the case for *Closterium* spp.). However, we argue that the clear longitudinal decrease of phytoplankton density from the lake outlet is convincing evidence that we are observing a dispersal pattern driven by a main propagule source.

On the biogeographical scale, different spatial units have been proposed to characterize stream ecosystems. The smallest spatial unit proposed for rivers is the site, around 10 m of river section (Frissell et al. 1986; Domisch et al. 2015). The next spatial unit is the reach (~100 m), which is defined as a river section lying between breaks in channel slope and local side slopes (Frissell et al. 1986; Domisch et al. 2015). Then, the *segment* (~5 km) has been proposed as the spatial river unit where homogeneous environmental conditions are found (Frissell et al. 1986; Domisch et al. 2015), and this unit is frequently applied in river classification systems (Snelder et al. 2005; Thorp et al. 2010). In this context, our study provides three new insights relevant for understanding stream algal metacommunity dynamics: (i) at the site scale, both self‐propelled dispersal (in motile algae) and flow‐mediated dispersal are likely to play important roles, making it difficult to differentiate between them; (ii) a reach section could be highly connected by algal migration, being long enough to contain several algal populations; and (iii) algal colonization across several segments is unlikely to occur in the environment of riffle‐pool streams, but this would be different in deeper rivers where longer dispersal trips should be frequent (Ock and Takemon 2010).

### 4.3 Advantages and Challenges of the Proposed Method

The most common approaches for simulating particle transport in streams focus on sediment transport, primarily for engineering purposes. These methodologies have advanced significantly, taking advantage of high computational power and becoming deeply integrated with geology and hydrography (Ancey 2020). The resulting complexity makes such tools challenging to apply to ecological questions at the population or community level. In contrast, our approach seeks to establish a direct relationship between habitat morphology and settling probability, thus enabling discussions of which habitat conditions influence the dispersal process driven by morphological and behavioral traits. This knowledge is particularly relevant for freshwater ecologists, as it provides a framework to contextualize micro‐scale dispersal processes within river morphology. Therefore, we consider that our modeling approach to be a significant contribution to current research on the topic. Future challenges for the suggested methodology include testing it across different stream types, for example by assessing how our habitat segmentation works in streams without an obvious pool‐riffle sequence, such as plane‐bed or dune‐ripple channels (Montgomery and Buffington 1997)—incorporating classical methodologies from fine particulate organic matter research to assess settling rates in situ (Cushing et al. 1993; Ock and Takemon 2010), and then comparing these with the values estimated by our model.

The last 20 years have seen a significant shift in how ecologists measure distance and track species movements, with tracking data becoming increasingly common and enabling more precise estimation of dispersal distances (Driscoll et al. 2014; Nathan et al. 2022). To analyze these datasets, fitting dispersal kernels is the most common strategy, summarizing movement data as a density function that describes the distance probability of species movements (Nathan et al. 2012; Proença‐Ferreira et al. 2023). Researchers typically fit parametric dispersal kernels such as Gaussian, exponential, power‐law, or 2Dt densities (Nathan et al. 2012). One of the key limitations of these parametric options is their inflexibility: characterizing dispersal patterns with uneven or “nonstandard” shapes using known probability distributions can be problematic. In our study, the data exhibited an almost linear decrease up to 1 km below the lake outlet and then flattened to a long right tail, albeit with minor local increases observed beyond 1 km for certain algal taxa. Nonparametric kernels captured the shape of our data while maintaining a smooth curve, allowing us to estimate dispersal distances that were aligned with field observations and provided insights into both the physical dispersal phenomena and habitat‐specific influences. We believe this approach represents a strong alternative for datasets with atypical distributions that do not conform to parametric dispersal kernels, even though it presents some new challenges. The primary concern lies in selecting the appropriate bandwidth; while data‐driven selectors exist, these can be unstable in some applications.

## 5 Conclusions

Our study developed and successfully tested a novel methodology to evaluate dispersal kernel distributions of stream periphyton. Moreover, our results provide two new insights into dispersal processes in lotic periphyton communities. First, potential dispersal distances for periphyton are likely to vary between 50 m and 1 km for pool‐riffle sequences in third‐ and fourth‐order stream reaches, with a low probability of much longer dispersal distances (> 2 km). Second, the considerable differences recorded in laboratory‐derived settling velocities between some of the studied taxa corresponded to relatively minor differences in potential travel distances in our study stream. This finding highlights the role of turbulence‐driven periphyton dispersal, which depends strongly on variability in morphology within the same algal taxon. The similarity between algal kernel distributions from tiles exposed in our study river and laboratory‐derived settling velocities demonstrates that our proposed methodology was successful in capturing periphyton dispersal processes. Future related research should determine how cellular density and hydraulic conditions interact to determine the long‐dispersal process of periphyton taxa, and whether changes in cellular density due to environmental conditions could be considered a migration mechanism in lotic periphyton.

## Author Contributions

**Daniel Zamorano:** conceptualization (lead), data curation (lead), formal analysis (equal), investigation (lead), methodology (lead), project administration (lead), software (equal), validation (equal), visualization (lead), writing – original draft (equal), writing – review and editing (supporting). **Claudio I. Meier:** formal analysis (supporting), investigation (equal), methodology (equal), software (supporting), validation (equal), writing – original draft (equal). **Tilman M. Davies:** formal analysis (supporting), investigation (equal), methodology (equal), software (supporting), validation (equal), writing – original draft (equal). **Travis Ingram:** conceptualization (supporting), formal analysis (supporting), investigation (supporting), validation (supporting), writing – original draft (supporting), writing – review and editing (supporting). **Ursula Romero:** conceptualization (supporting), investigation (supporting), methodology (supporting), supervision (supporting), validation (supporting). **Christoph D. Matthaei:** conceptualization (supporting), formal analysis (supporting), funding acquisition (lead), investigation (equal), methodology (equal), project administration (supporting), supervision (lead), writing – original draft (equal), writing – review and editing (lead).

## Funding

This work was supported by a PhD research expense grant, Department of Zoology, University of Otago.

## Conflicts of Interest

The authors declare no conflicts of interest.

## Supporting information


**Figure S1:** Feeding status and desiccation tolerance in honey bees. This pilot study assessed the effect of food access on survival time under simulated acute desiccation stress, water loss rate (WLC, % h^−1^), and critical water content (CWC, %). (a) Comparison fed (*n* = 13) and unfed (*n* = 12) bees in (a) initial body mass, (b) survival time, (c) water loss rate, and (d) critical water content. Box plots show median, quartiles, and extreme values. For each figure, a different letter above bars indicates significant differences (*p <* 0.05).
**Figure S2:** Alluvial plot showing the associations among nesting guilds, sociality, and nest location. The width of each stream represents the frequency of species sharing the same combination of ecological traits. Strata represent individual trait categories, while alluvia illustrate the flow of trait combinations across axes. Colors indicate nesting guilds. Nesting guild: Ground = strictly ground nesters; Hole = nesting in pre‐existing cavities or constructed holes; Cavity = large comb‐building cavity nesters. Nest location: below ground, above ground or both. Social behavior: solitary, eusocial, and socially polymorphic species.
**Figure S3:** Pairwise plot matrix showing the distribution and relationships among model variables. Density plots are displayed along the diagonal, scatterplots in the lower left, and correlations coefficients in the upper right. Variables: Survival = bee survival time (hours); WLR = water loss rate (% h^−1^); CWC = critical water content (%); FreshMass = wet mass (g); DryMass = dry body mass (g); HairLen = hair length (mm).
**Figure S4:** Pairwise plot matrix showing the distribution and relationships among log_10_‐transformed and untransformed variables. Density plots are displayed along the diagonal, scatterplots in the lower left, and correlations coefficients in the upper right. Variables: L10Survival = Log_10_‐transformed bee survival time (hours); L10WLR = Log_10_‐transformed water loss rate (% h^−1^); ArcCWC = arcsine transformed critical water content (%); L10FreshMass = Log_10_‐transformed wet mass (g); L10DryMass= Log_10_‐transformed dry body mass (g); HairLen= hair length (mm).
**Table S1:** Life‐history, morphological, and desiccation‐related traits of bee species used in desiccation tolerance assays from the Greek island of Lesbos. Nesting guild (NG): A = aerial; G = ground‐nesters; H = hole‐nesters; L = Large cavity nesters. Nest location (NL): AB = above ground; BG = below ground; B = both. Sociality: E = Eusocial; *P* = socially polymorphic; S = solitary.
**Table S2:** Comparison of linear mixed models assessing the influence of body mass and hair length as predictors of water loss rate and critical water content across 22 bee species. For each response variable, we fitted models initially including each morphological trait independently, then expanding them to include both morphological traits and their interactions. Model comparison using Akaike's Information Criterion (AIC) was used to select the best‐fitting model for each response variable. Best models and significant effects are indicated in bold (*p* < 0.05). Species and collection date were used as random effects.
**Table S3:** Results of the linear mixed‐effects model assessing for differences in survival time under desiccating conditions, water loss rate (WLR), and critical water content (CWC) among nesting guild, nest location, and sociality. This table presents the estimated coefficients, standard errors, *t*‐values, and *p*‐values for the fixed effects in models analyzing the response variables: Duration, Water Loss Rate (WLR), and Critical Water Content (CWC). Factors such as Nesting, Nest Location, and Sociality were included in the models, with results for each factor provided.
**Table S4:** Number of individual bees and species represented in each nesting guild, nest location, and sociality group included in the principal component analysis. See also Figure 2.
**Table S5:** Results of the linear mixed‐effects model predicting survival time under desiccating conditions in 15 bee species as a function of water loss rate (Log_10_WLR), critical water content (ArcCWC), and body wet mass (Log_10_Mass). In these analyses, seven species represented by a single specimen were excluded. The final best‐fitting model included all fixed effects and a single interaction, as indicated below. Species and collection date were included as random effects.
**Table S6:** Comparison of linear mixed models assessing the influence of body mass and hair length as predictors of water loss rate and critical water content across 15 bee species. In these analyses, seven species represented by a single specimen were excluded. For each response variable, we fitted models initially including each morphological trait independently, then expanding them to include both morphological traits and their interactions. Model comparison using Akaike's Information Criterion (AIC) was used to select the best‐fitting model for each response variable. Best models and significant effects are indicated in bold (*p* < 0.05). Species and collection date were used as random effects.
**Table S7:** Results of the linear mixed‐effects model assessing for differences in survival time under desiccating conditions, water loss rate (WLR), and critical water content (CWC) among nesting guild, nest location, and sociality across 15 species. In these analyses, seven species represented by a single specimen were excluded. This table presents the estimated coefficients, standard errors, *t*‐values, and *p*‐values for the fixed effects in models analyzing the response variables: Survival time, Water Loss Rate (WLR), and Critical Water Content (CWC). Factors such as Nesting, Nest Location, and Sociality were included in the models, with results for each factor provided.

## Data Availability

The raw data of this study, including dispersal distances and settling‐velocity data, plus the R code with all models and graphs, were included as an online supplement at the following link: https://doi.org/10.6084/m9.figshare.24223693.
