# Supplementary material for: Estimating Dispersal Kernels for Stream Periphyton Using Drifting Lake Algae
Source: Ecol Evol. 2026 Sep 13;16(9):e74334. doi: 10.1002/ece3.74334 (PMC13572789; doi:10.1002/ece3.74334)
Supplement: Supplementary file 1 [file ECE3-16-e74334-s001.docx]

Supplementary Material

**Estimating dispersal kernels for stream periphyton using drifting lake algae**

Daniel Zamorano ^1*^ (ORCID ID: 0000-0001-8235-3990)

Claudio I. Meier ^2^ (ORCID ID: 0000-0002-5918-6001)

Tilman M. Davies ^3^ (ORCID ID: 0000-0003-0565-1825)

Travis Ingram ^1^ (ORCID ID: 0000-0003-0709-5260)

Ursula Romero ^4^

Christoph D. Matthaei ^1^ (ORCID ID: 0000-0002-4823-9198)

^1^ Department of Zoology, University of Otago, Dunedin, New Zealand.

^2^ Department of Civil Engineering, University of Memphis, Memphis, TN, USA.

^3^ Department of Mathematics & Statistics, University of Otago, Dunedin, New Zealand.

^4^ Laboratorio de Limnología, Facultad de Ciencias, Universidad de Chile, Santiago, Chile.

[Appendix S1	3](#_Toc198722485)

[Statistical Hydraulic model details	3](#_Toc198722486)

[Rouse number assumptions	3](#_Toc198722487)

[Computations for the riffles	4](#_Toc198722488)

[Computations for the pools	6](#_Toc198722489)

[River parameters	6](#_Toc198722490)

[Simulations	7](#_Toc198722491)

[Appendix S2	9](#_Toc198722492)

[Details on Kernel density estimation and its implementation	9](#_Toc198722493)

[Methodology	9](#_Toc198722494)

[Univariate KDE	9](#_Toc198722495)

[Bandwidth selection and boundary correction	10](#_Toc198722496)

[Bootstrapping for confidence bands	11](#_Toc198722497)

[R functionality for this analysis	11](#_Toc198722498)

[Required package loadings	11](#_Toc198722499)

[Establishing parallelization parameters	11](#_Toc198722500)

[Custom KDE function	12](#_Toc198722501)

[A quick example using the evmix R package	12](#_Toc198722502)

[Real-world data	14](#_Toc198722503)

[Discretized distance traps and jittering	15](#_Toc198722504)

[Implementation of KDE and finding confidence ‘extremes’	17](#_Toc198722505)

[Settling-velocity experiment data	20](#_Toc198722506)

[Figure S1	22](#_Toc198722507)

[Figure S2	23](#_Toc198722508)

[Figure S2a	23](#_Toc198722509)

[Figure S2b	24](#_Toc198722510)

[Table S1	35](#_Toc198722511)

[Table S2	26](#_Toc198722512)

[Table S3	28](#_Toc198722513)

[Table S4	30](#_Toc198722514)

[Table S5	34](#_Toc198722515)

[References	43](#_Toc198722516)

# Appendix S1

## Statistical Hydraulic model details

### Rouse number assumptions

By definition, at any point inside a turbulent flowing field, there is a turbulent shear stress with a mean value $\boldsymbol{\tau}$, which depends on the location within the 3-D flow. At that point, the shear velocity ($\boldsymbol{u}_{\boldsymbol{*}}$) is given by:

$\boldsymbol{u}_{\boldsymbol{*}}= \sqrt{\frac{\boldsymbol{\tau}}{\rho}}$	(1)

Where $\boldsymbol{\rho}$ is the density of the fluid.

Because we adopted a 1-D model, we based our approach on whole-channel and whole-flow descriptions. In such cases, the shear velocity was evaluated based on the mean shear stress at the bed of the open channel, $\boldsymbol{\tau}_{\boldsymbol{0}}$:

$\boldsymbol{u}_{\boldsymbol{*}}= \sqrt{\frac{\boldsymbol{\tau}_{\boldsymbol{0}}}{\rho}}$	(2)

In a real channel, the bed (aka boundary) shear stress changes spatially. In our 1-D representation, we introduced two simplifications to deal with this:

1. We assumed that the flow is uniform along each reach habitat, so the flow depth is the same everywhere, as is the flow velocity. Note here that we only were able to assume fully uniform flow in our riffles. For the pools we assumed a flat bed (i.e., pools that are rectangular parallelepipeds), so the flow cannot be exactly uniform (as that would imply a flat free surface, and thus no flow). However, given the low magnitude of the free-surface slope, and the fact that we model low discharges, the flow can be considered to be quasi uniform (sensu Dingman, 2009, p. 215).
2. Related to this, we neglected lateral (or transversal) variations in the cross-section, and we also assumed that the effects of the banks can be “averaged out,” by using mean velocities and mean bed shear stresses. In other words, our riffles and pools are all rectangular, so the depth is the same everywhere across the section.

Of course, it should be clear that the flow in a real-world river would not be uniform longitudinally. Even in a “well-behaved” flow (technically called a “gradually varied flow”), assuming riffles with uniform characteristics (such as longitudinal slope ***S_0_***, width ***b***, etc.), the flow profile will tend to be uniform only in the middle reach of the riffle, away from its extremes. But flow depths and velocities will trend away from the uniform case when the riffle flow decelerates as it plunges into the next pool, as well as immediately below the previous pool, where the water accelerates into the riffle. Still, these reaches with appreciable acceleration and deceleration should be rather short, so the assumption should not introduce much of a bias.

Neither is the flow uniform transversally. In the real river with 3-D flow, the flow depths, velocities, and turbulent fluctuations will be highly reduced when coming closer to the shoreline, so that any particle able to find its way there will have much increased chances for settling. Our 1-D modeling framework basically neglected this, as it “averages it out” across a rectangular, rather wide section.

The mean bed shear stress is given by:

$\boldsymbol{\tau}_{\boldsymbol{0}}= \rho g \boldsymbol{R}_{\boldsymbol{h}} \boldsymbol{S}_{\boldsymbol{f}}$	(3)

where $\rho$ is the density of water (1000 kg/m^3^), *g* = 9.81 m/s^2^, $\boldsymbol{R}_{\boldsymbol{h}}$ is the hydraulic radius defined as

$\boldsymbol{R}_{\boldsymbol{h}}= \frac{\boldsymbol{A}}{\boldsymbol{P}}$	(4)

where, in turn, $\boldsymbol{A}$ is the cross-sectional area of flow (m^2^) while $\boldsymbol{P}$ is the wet perimeter (m), which can be computed for a known cross-sectional geometry (i.e., shape) if the flow depth is known, just assuming that the free surface is horizontal (as done in 1-D hydraulics; typically, a very good assumption). $\boldsymbol{S}_{\boldsymbol{f}}$is known as the friction slope, which represents the local rate of energy dissipation in the longitudinal direction (i.e., how much hydraulic head in m is lost as the water flows 1 m in the downstream direction). In the case of uniform flow in the riffles, we can replace $\boldsymbol{S}_{\boldsymbol{f}}$by the riffle bed slope $\boldsymbol{S}_{\boldsymbol{0}}$, while in the case of flow in the pools, we will need to compute the actual value of $\boldsymbol{S}_{\boldsymbol{f}}$, using Gauckler-Manning’s equation (see Eq. 15 below).

### Computations for the riffles

Each riffle will have a certain length $\boldsymbol{L}_{\boldsymbol{r}}$, a certain width $\boldsymbol{b}_{\boldsymbol{r}}$, a certain bed slope $\boldsymbol{S}_{\boldsymbol{0}\boldsymbol{r}}$, and a certain roughness, as given by a Manning’s $\boldsymbol{n}_{\boldsymbol{r}}$. Riffle lengths were always integer multiples of the channel width. Given a known, assumed discharge ***Q*** for the simulation, we wrote the Gauckler-Manning’s (G-M) equation as follows:

(5) $\boldsymbol{Q=}\frac{\boldsymbol{1}}{\boldsymbol{n}}\boldsymbol{A}\left( \boldsymbol{y}_{\boldsymbol{r}} \right) \left[ \boldsymbol{R}_{\boldsymbol{h}}\left( \boldsymbol{y}_{\boldsymbol{r}} \right) \right]^{\frac{\boldsymbol{2}}{\boldsymbol{3}}} \sqrt{\boldsymbol{S}_{\boldsymbol{0}\boldsymbol{r}}}$

In this equation, the discharge $\boldsymbol{Q}$, the riffle roughness $\boldsymbol{n}_{\boldsymbol{r}}$, and the riffle slope $\boldsymbol{S}_{\boldsymbol{0}\boldsymbol{r}}$ are known (because they are assumed before each simulation). What is solved is the as-of-yet unknown flow depth in the riffle, $\boldsymbol{y}_{\boldsymbol{r}}$. For a rectangular riffle of known width $\boldsymbol{b}_{\boldsymbol{r}}$, the flow area will be given by:

$\boldsymbol{A}\left( \boldsymbol{y}_{\boldsymbol{r}} \right)=\boldsymbol{b}_{\boldsymbol{r}} \cdot\boldsymbol{y}_{\boldsymbol{r}}$	(6)

Because we already neglected lateral variation and considered rectangular channels that are quite much wider than the flow depth, we further assume that the hydraulic radius (“the mean distance from the free surface to the nearest boundary”) is equal to the flow depth: $\boldsymbol{R}_{\boldsymbol{h}}=\boldsymbol{y}_{\boldsymbol{r}}$.

Under this simplification, the G-M equation becomes:

$\boldsymbol{Q} = \frac{1}{\boldsymbol{n}_{\boldsymbol{r}}} \boldsymbol{y}_{\boldsymbol{r}}^{\frac{5}{3}} \boldsymbol{b}_{\boldsymbol{r}} \sqrt{\boldsymbol{S}_{\boldsymbol{0}\boldsymbol{r}}}$	(7)

wherefrom the flow depth in the riffle can be solved from known values as follows

$\boldsymbol{y}_{\boldsymbol{r}}= \left( \frac{\boldsymbol{Q} \boldsymbol{n}_{\boldsymbol{r}}}{\boldsymbol{b}_{\boldsymbol{r}} \sqrt{\boldsymbol{S}_{\boldsymbol{0}\boldsymbol{r}}}} \right)^{\frac{3}{5}}$	(8)

Now, replacing the value of $\tau_{0}$ (Eq. 3) into the expression for the shear velocity $\boldsymbol{u}_{\boldsymbol{*r}}$ in the riffle (Eq. 2):

$\boldsymbol{u}_{\boldsymbol{*r}}= \sqrt{\frac{\boldsymbol{\tau}_{\boldsymbol{0}}}{\rho}}= \sqrt{\frac{\rho g \boldsymbol{R}_{\boldsymbol{h}} \boldsymbol{S}_{\boldsymbol{f}}}{\rho}}= \sqrt{g \boldsymbol{R}_{\boldsymbol{h}} \boldsymbol{S}_{\boldsymbol{f}}}$	(9)

In our latest simplification we assumed that $\boldsymbol{R}_{\boldsymbol{h}}=\boldsymbol{y}_{\boldsymbol{r}}$, and for the riffles we have that the friction slope $\boldsymbol{S}_{\boldsymbol{f}}$(slope of the energy line) is the same as the longitudinal bed slope $\boldsymbol{S}_{\boldsymbol{0}\boldsymbol{r}}$, as we are assuming uniform flow. Thus, the shear velocity is given by:

$\boldsymbol{u}_{\boldsymbol{*r}}= \sqrt{g \boldsymbol{R}_{\boldsymbol{h}} \boldsymbol{S}_{\boldsymbol{f}}}= \sqrt{9.81 \frac{m}{s^{2}} \cdot\boldsymbol{y}_{\boldsymbol{r}} \cdot\boldsymbol{S}_{\boldsymbol{0}\boldsymbol{r}}}$	(10)

Where the flow depth $\boldsymbol{y}_{\boldsymbol{r}}$ is in meters, while the slope $\boldsymbol{S}_{\boldsymbol{0}\boldsymbol{r}}$ is in proportion of 1. Having computed $\boldsymbol{u}_{\boldsymbol{*r}}$ we now obtain the Rouse number for the riffle as:

$\boldsymbol{R}_{\boldsymbol{o riffle}}= \frac{\boldsymbol{V}_{\boldsymbol{s}}}{\kappa\boldsymbol{u}_{\boldsymbol{*r}}}$	(11)

where $\kappa=0.40$ is the dimensionless von Kármán constant.

### Computations for the pools

In turn, each pool was a rectangular parallelepiped having a certain length $\boldsymbol{L}_{\boldsymbol{p}}$, a certain width $\boldsymbol{b}_{\boldsymbol{p}}$, a certain depth $\boldsymbol{d}_{\boldsymbol{p}}$, and a certain roughness, as given by a Manning’s $\boldsymbol{n}_{\boldsymbol{p}}$. Pool lengths were integer multiples of the pool width. The flow depth in a given pool ***i*** was computed as the depth of the pool $\boldsymbol{d}_{\boldsymbol{p} i}$ plus the flow depth $\boldsymbol{y}_{\boldsymbol{r} i}$ in the riffle immediately below it (which has the same generic numbering ***i***):

$\boldsymbol{y}_{\boldsymbol{p} i}= \boldsymbol{d}_{\boldsymbol{p} i}+ \boldsymbol{y}_{\boldsymbol{r} i}$	(12)

Given the flow depth in a pool $y_{p}$, we computed its cross-sectional area and wet perimeter as:

$\boldsymbol{A}\left( \boldsymbol{y}_{\boldsymbol{p}} \right)=\boldsymbol{b}_{\boldsymbol{p}} \cdot\boldsymbol{y}_{\boldsymbol{p}}$	(13)

and

$\boldsymbol{P}\left( \boldsymbol{y}_{\boldsymbol{p}} \right)=\boldsymbol{b}_{\boldsymbol{p}}+ 2\cdot\boldsymbol{y}_{\boldsymbol{p}}$	(14)

And we solved G-M’s equation for the friction slope:

$\boldsymbol{Q}= \frac{1}{\boldsymbol{n}_{\boldsymbol{p}}} \frac{\left[ \boldsymbol{A}\left( \boldsymbol{y}_{\boldsymbol{p}} \right) \right]^{\frac{5}{3}}}{\left[ \boldsymbol{P}\left( \boldsymbol{y}_{\boldsymbol{p}} \right) \right]^{\frac{2}{3}}} \sqrt{\boldsymbol{S}_{\boldsymbol{f}}} \Longrightarrow\boldsymbol{S}_{\boldsymbol{f}}= \left( \frac{\boldsymbol{Q} \boldsymbol{n}_{\boldsymbol{p}} \left[ \boldsymbol{P}\left( \boldsymbol{y}_{\boldsymbol{p}} \right) \right]^{\frac{2}{3}}}{\left[ \boldsymbol{A}\left( \boldsymbol{y}_{\boldsymbol{p}} \right) \right]^{\frac{5}{3}}} \right)^{2} = \frac{\boldsymbol{Q}^{2} \boldsymbol{n}_{\boldsymbol{p}}^{2} \left[ \boldsymbol{P}\left( \boldsymbol{y}_{\boldsymbol{p}} \right) \right]^{\frac{4}{3}}}{\left[ \boldsymbol{A}\left( \boldsymbol{y}_{\boldsymbol{p}} \right) \right]^{\frac{10}{3}}}$	(15)

Allowing us to compute the shear velocity in the pool:

$\boldsymbol{u}_{\boldsymbol{*p}}= \sqrt{g \boldsymbol{R}_{\boldsymbol{h}} \boldsymbol{S}_{\boldsymbol{f}}}= \sqrt{9.81 \frac{m}{s^{2}} \cdot\frac{\boldsymbol{A}\left( \boldsymbol{y}_{\boldsymbol{p}} \right)}{\boldsymbol{P}\left( \boldsymbol{y}_{\boldsymbol{p}} \right)} \cdot\boldsymbol{S}_{\boldsymbol{f}}}$	(16)

Where the flow area $\boldsymbol{A}$ is in meters squared, the wet perimeter $\boldsymbol{P}$ in meters, while the slope $\boldsymbol{S}_{\boldsymbol{f}}$ is again in proportion of 1. Having computed $\boldsymbol{u}_{\boldsymbol{*p}}$, the shear velocity in the pool, we obtain the Rouse number for the pool as:

$\boldsymbol{R}_{\boldsymbol{o pool}}= \frac{\boldsymbol{V}_{\boldsymbol{s}}}{\kappa\boldsymbol{u}_{\boldsymbol{*p}}}$	(17)

### River parameters

The following parameters were fitted to characterize the river’s morphology in the simulations: pool and riffle width ($\boldsymbol{b}_{\boldsymbol{p}}$ and $\boldsymbol{b}_{\boldsymbol{r}}$), pool and riffle roughness ($\boldsymbol{n}_{\boldsymbol{p}}$ and $\boldsymbol{n}_{\boldsymbol{r}}$), pool depth ($\boldsymbol{d}_{\boldsymbol{p}}$), riffle slope ($\boldsymbol{S}_{\boldsymbol{0}\boldsymbol{r}}$) and the river flow ($\boldsymbol{Q}$).

Since this study focuses on algae dispersal in a given river, with an emphasis on algal morphology, we set these parameters looking for an average representation of the river studied. Thus, we randomized river parameters to represent the channel’s variability, but in a range that still reflected a standard representation of the river. Pool and riffle widths were randomly generated using a gamma distribution (shape = 22.5, rate = 0.066), with a right-skewed distribution. We obtained an average width of 1.5 m and a standard deviation of 0.31 m, consistent with field observations.

Pool and riffle roughness values were randomly generated using a normal distribution ($\boldsymbol{n}_{\boldsymbol{p}}$: x̄ **=** 0.04, σ = 0.004; $\boldsymbol{n}_{\boldsymbol{r}}$: x̄ **=** 0.18, σ = 0.018). Roughness values were chosen based on roughness measurements observed in similar rivers in New Zealand (Hicks 1991). Pool depths were randomly generated using a normal distribution ($\boldsymbol{d}_{\boldsymbol{p}}$: x̄ **=** 0.3, σ = 0.003), consistent with field observations. Riffle slopes were randomly generated using a normal distribution ($\boldsymbol{S}_{\boldsymbol{0}\boldsymbol{r}}$: x̄ **=** 0.55, σ = 0.0055), with values fitted to match elevation changes along the river, as derived from the New Zealand 8m DEM (2012, [link](https://data.linz.govt.nz/layer/51768-nz-8m-digital-elevation-model-2012/metadata/iso/)). Finally, river flow was held constant in all simulations (0.12 m^3^/s), to explore algal dispersal under constant flow conditions. The river flow was determined based on flow records obtained by Otago Regional Council at the river mouth of the Water of Leith using simple scaling by catchment area ([link](https://envdata.orc.govt.nz/AQWebPortal/Data/Location/Dashboard/405/Location/EY199/Interval/Latest)), which is applicable for low flows.

### Simulations

Simulations were run until algal settling occurred, applying the equation explained in the manuscript’s method section. The simulation process consisted of the following sequential steps:

1. The morphological characteristics for one pool and one riffle were determined using the randomization algorithms explained earlier. These included pool and riffle width ($\boldsymbol{b}_{\boldsymbol{p}}$ and $\boldsymbol{b}_{\boldsymbol{r}}$), pool and riffle roughness ($\boldsymbol{n}_{\boldsymbol{p}}$ and $\boldsymbol{n}_{\boldsymbol{r}}$), pool depth ($\boldsymbol{d}_{\boldsymbol{p}}$), riffle slope ($\boldsymbol{S}_{\boldsymbol{0}\boldsymbol{r}}$). River flow was set beforehand. Additionally, it was randomly and independently determined whether the pool and riffle lengths would be 2, 3, or 4 times the pool and riffle widths, respectively.
2. Using the selected values, key flow variables were estimated: riffle water depth ($\boldsymbol{y}_{\boldsymbol{r}}$), pool and riffle cross-sectional areas and wet perimeters ($\boldsymbol{A}\left( \boldsymbol{y}_{\boldsymbol{p}} \right)$, $\boldsymbol{P}\left( \boldsymbol{y}_{\boldsymbol{p}} \right)$, $\boldsymbol{A}\left( \boldsymbol{y}_{\boldsymbol{r}} \right)$, and $\boldsymbol{P}\left( \boldsymbol{y}_{\boldsymbol{r}} \right)$), and friction slope ($\boldsymbol{S}_{\boldsymbol{f}}$). Considering these values, the Rouse numbers were estimated for the pool and the riffle ($\boldsymbol{R}_{\boldsymbol{o pool}}$ and $\boldsymbol{R}_{\boldsymbol{o riffle}}$).
3. Each habitat length was subdivided into 3 sections per channel width. For example, if the habitat length was randomly chosen to have 3 times the channel width, we had 9 sections per habitat. These sections were arranged sequentially (upstream to downstream) along the habitat. For each section, a settling probability was estimated.
4. The first habitat evaluated is the pool, followed by the riffle. If the algae did not settle, another pool-riffle habitat pair was randomly generated. Travel distance was estimated by summing the lengths of habitat sections where the algae did not settle (with each section being one-third of the channel width).

This algorithm was run 1000 times per algal taxon, using algae settling velocities generated randomly from a lognormal distribution fitted to their settling velocities observed in laboratory experiments.

# Appendix S2

## Details on Kernel density estimation and its implementation

### Methodology

In many ecological applications, dispersion kernels are estimated using well-known parametric probability distributions (Nathan et al. 2012), such as those derived from the exponential family. This is convenient, since we need only estimate a small number of parameters (typically just one or two) from the sampled data of traveled distances. In cases where the empirical distribution of the observed distances shows significant departures from one of the commonly-used parametric distributions, more flexible estimation methods may be appealing. For this reason, we turned to *kernel density estimation* (KDE) in the analysis of the data collected as part of this research.

This document aims to provide a brief overview of KDE and important topics associated with its implementation, supplementing the main paper. We then demonstrate how it is applied to the data arising from the current study.

### Univariate KDE

Kernel density estimation is a nonparametric smoothing method used to estimate the probability distribution of a set of observations. It is highly flexible and is therefore useful when the density of interest is complex or does not adhere to one of the more common ‘known’ parametric distributions, such as the normal distribution. The trade-off lies in the amount of smoothing we choose to use, with a greater amount of smoothing leading to a more heavily biased density estimate, and a smaller degree of smoothing limiting this bias at the expense of increasing variability/noise in the final estimate.

Assume the presence of $n$ univariate observations $\{x_{1},\ldots,x_{n}\}$ arising from some unknown density function $f$. The kernel density estimate of $f$, $\hat{f}_{h}$, is given by the following equation:

$$\hat{f}_{h}\left( x \right)=\frac{1}{nh}\sum_{i=1}^{n} K\left( x|x_{i};h \right)=\frac{1}{nh}\sum_{i=1}^{n} K\left( \frac{x-x_{i}}{h} \right).$$

Here, $h>0$ is the *bandwidth* or *smoothing parameter*, while $K\left( \cdot\right)$ is the *kernel function* (this is not to be confused with the use of the term “kernel” in “dispersion kernel”). The latter is typically chosen to be a zero-mean, symmetric probability density function such as the normal distribution, which is what we use here.

### Bandwidth selection and boundary correction

The bandwidth $h$ is of the utmost importance in producing $\hat{f}_{h}$. Various data-driven methods exist to choose an ‘optimal’ amount of smoothing—a value of $h$ that is neither ‘too small’ nor ‘too big’—all of which have benefits and drawbacks in terms of theoretical properties and practical performance. In this work, we focus on a method known as *biased cross-validation* to choose $h$. This method seeks to optimise the bandwidth as an appropriate balance between the bias and variance of the result, using asymptotic approximations to these quantities. It is chosen as it tends to be more stable and offer slightly more smoothing, on average, than some alternative selectors. We found this behavior to be beneficial in the current application.

Another important issue in implementing KDE is *boundary correction*. This arises when smoothing data near boundaries of the domain. Specifically, in the current application we have a hard ‘boundary’ at a distance of zero, that is, it is not possible to observe negative distances $x$. As such, we need to correct for the ‘weight’ of the kernel function $K$ in the expression for $\hat{f}_{h}\left( x \right)$ that is lost over the boundary at zero for values $x<0$. Ignoring this leads to poor estimation of the distribution of traveled distances for small $x>0$.

As with bandwidth selection, there are various ways to adjust for these unsavory boundary effects. In this work we use the ‘reflection’ approach (Jones 1993), which, for a hard boundary at $x=0$, consists of replacing $K\left( x|x_{i};h \right)$ in the above with

$$\bar{K}\left( x|x_{i};h \right)=K\left( \frac{x-x_{i}}{h} \right)+K\left( \frac{x+x_{i}}{h} \right).$$

This has the effect of reflecting the kernel weight that would be lost over the boundary (i.e. for values $x<0$) in the uncorrected version back on to the positive side, mitigating the negative bias that would otherwise occur.

For more details on kernel density estimation, the texts by Wand and Jones (1994), Bowman and Azzalini (1997), and Silverman (2017) contain good introductions for interested readers.

### Bootstrapping for confidence bands

It is of interest to gauge the uncertainty associated with a given density estimate. A simple way to do so is to *bootstrap* the observations (resample with replacement) and re-estimate the density, repeating this many times. Approximate ‘confidence bands’ may then be found via the corresponding quantiles. Specifically, for the observed data $X=\{x_{1},\ldots,x_{n}\}$, and associated density estimate $\hat{f}_{h}$ the steps are as follows:

1. Resample $n^{*}$ observations from $X$, with replacement, giving $X^{*}=\{x_{1}^{*},\ldots,x_{n^{*}}^{*}\}$. Typically, $n^{*}=n$.
2. Estimate the density of $X^{*}$ via KDE, giving $\hat{f}_{h}^{*}\left( x \right)$ for values of $x$ over the assumed domain of $X$. Retain this estimate.
3. Repeat steps 1-2 $M$ times.
4. For all values $x$ of interest, find the $\left( \alpha/2 \right)$th and $\left( 1-\alpha/2 \right)$th quantiles of the set of $M$ bootstrapped density estimates to compute the confidence bands; $0\leq\alpha\leq1$ is the corresponding significance level (e.g. set $\alpha=0.05$ for 95% confidence bands).

## R functionality for this analysis

### Required package loadings

library("evmix",quietly=TRUE) # used for boundary-corrected univariate KDE
library("doParallel",quietly=TRUE) # used to access multiple CPU cores for faster completion

### Establishing parallelization parameters

Of note is that any parallel computation executed in this report is system-dependent. We will define a constant value, PARA, to govern this. To find the total number (physical and virtual) of CPU cores available to you, note the following result:

```
totcores <- detectCores()
totcores
## [1] 16
```

(If you are not knitting the .Rmd file of this document on your own machine, and instead reading this in a finalized form, the above results of course hold only for the machine on which it was originally processed.)

A good rule-of-thumb, when running on an otherwise idle system, is to set PARA to be 2 less than totcores:

```
PARA <- totcores-2
PARA
## [1] 14
```

Note that running parallel computations is power-hungry; ensure a sufficiently charged battery (or ready access to mains power) if knitting this document on a laptop. To avoid parallel computation, set PARA <- 1.

### Custom KDE function

In Sections 3 and 4 of this report, we will make use of a custom function named kdeboot. This is created here and its role will be explained later.

```
kdeboot <- function(xv,X,hb,K=100,para=1,size=length(X)){
  if(para<=1){
    boots <- replicate(K,{
      Xs <- sample(X,replace=TRUE)
      fboot <- dbckden(xv,Xs,bw=hb,bcmethod="reflect")
    })
  } else {
    totcor <- parallel::detectCores()
    if(para>totcor) stop(paste("your machine can only cope with a maximum 'para' value of",torcor))
    registerDoParallel(cores=para)
    boots <- foreach(i=1:K,.packages="evmix",.combine=cbind) %dopar% {
      set.seed(runif(1,1,10000))
      Xs <- sample(X,size=size,replace=TRUE)
      fboot <- dbckden(xv,Xs,bw=hb,bcmethod="reflect")
      qboot <- qbckden(c(0.01,0.025,0.05,0.1,0.25,0.5,0.75,0.9,0.95,0.975,0.99),Xs,bw=hb,bcmethod="reflect")
      return(c(fboot,qboot))
    }
  }
  result <- apply(boots,1,quantile,prob=c(0.025,0.975))
  return(result)
}
```

### A quick example using the evmix R package

The basics of the approach taken in this work are initially explored via an illustrative example.

First, consider the 272 observations of the faithful dataset, detailing eruption durations, in seconds, of the Old Faithful geyser in Yellowstone National Park, USA:

X <- faithful$eruptions
hist(X)

The following code sets up a sequence of values over which to compute a kernel density estimate of the eruption data (xvals), finds an appropriate smoothing bandwidth using biased cross-validation (h), uses dbckden from the evmix package to calculate the density estimate itself (fhat), and plots the result:

xvals <- seq(1,5.5,length=512)
h <- bw.bcv(X)
fhat <- dbckden(xvals,X,bw=h,bcmethod="reflect")
plot(xvals,fhat,type="l",xlab="eruption duration")

If we also want to find a bootstrap confidence interval to gauge uncertainty in the density estimate, the following code uses parallel computations (via the foreach function) to sample repeatedly (M times) with replacement, evaluate the corresponding bootstrapped density estimate (fboot), and store all M results over all values in the domain (xvals) as a length(xvals) $\times$ M matrix (fboots):

```
M <- 100
registerDoParallel(cores=PARA)
fboots <- foreach(i=1:M,.packages="evmix",.combine=cbind) %dopar% {
  set.seed(runif(1,1,10000))
  Xs <- sample(X,size=length(X),replace=TRUE)
  fboot <- dbckden(xvals,Xs,bw=h,bcmethod="reflect")
  return(fboot)
}
```

[[Approx. execution time for preceding code chunk: 8.63 seconds]].

We can then replot the density estimate from earlier and superimpose a corresponding 95% confidence interval derived from the appropriate quantiles of the results in fboots using the following:

confbands <- apply(fboots,1,quantile,probs=c(0.025,0.975))
plot(xvals,fhat,type="l",xlab="eruption duration",ylim=range(confbands),lwd=2)
lines(xvals,confbands[1,],lty=2,col=2)
lines(xvals,confbands[2,],lty=2,col=2)

## Real-world data

We will use the ideas illustrated in the preceding example in producing estimates of the dispersal distance densities for the algal taxa in our study. The phytoplankton density dataset has some complicating features. We begin this section by describing these features and our strategies for handling them, before illustrating how the results in the main paper were derived. We read in the following data frame, storing a vector of character strings defining the columns of the observations of interest:

```
dfalgae <- read.csv("dfalgae.csv")
sp_list <- c("Closterium_spp","Euglena_spp","Dictyosphaerium_spp", "Flagellate_spp","Peridinium_spp",
             "Cryptomonas_spp","Phacus_spp", "Chlamydomonas_spp","Monoraphidium_spp","Trachelomonas_spp","lake_sp")
```

### Discretized distance traps and jittering

The data are comprised of trapped taxon counts read off tiles placed at each of 20 discrete distance positions. These are sufficiently separate such that producing a continuous density estimate is unreasonable, as the duplication of the data at specific distances is extreme. We therefore consider each tile to ‘own’ the distances to the halfway mark of the separation between itself and the next distance position. This defines a series of intervals/zones within which we will uniformly jitter a proportionate number of observations prior to implementing KDE. This will respect the relative position-specific algal counts, at least approximately, and overcome the problem of density estimation with heavily tied observations.

The following code creates the object jvals, a data frame containing each unique tile distance value and its corresponding jittering zone:

```
dist <- dfalgae$Distance/100
unidist <- sort(unique(dist))
udl <- length(unidist)
jvals_left <- jvals_right <- c()
for(i in 1:udl){
  if(i<udl){
    jvals_right <- c(jvals_right,(unidist[i+1]-unidist[i])/2)
  }
  if(i>1){
    jvals_left <- c(jvals_left,(unidist[i]-unidist[i-1])/2)
  }
}
jvals <- data.frame(left=c(jvals_left[1],jvals_left),dist=unidist,right=c(jvals_right,jvals_right[udl-1]))
```

Using jvals, we may also create the following plot to visualize the design. Herein the y-axis denotes the specific distance of each tile with dots, and the encapsulating parentheses represent the distances that the corresponding tile ‘owns’ for the purposes of jittering:

```
plot(1:udl,unidist,cex=0.5,ylim=range(jvals[,2]-jvals[,1],jvals[,2]+jvals[,3]),
     xlab="position",ylab="distance",main="Jittering zones")
for(i in 1:udl){
  text(i,unidist[i]-jvals[i,1],"(",srt=90)
  text(i,unidist[i]+jvals[i,3],")",srt=90)
  abline(h=unidist[i]+jvals[i,3],col="darkgrey",lty=2)
}
```

We can illustrate the effect of jittering using these zones on the *Closterium* spp. data with the following example (column "Closterium_spp" in dfalgae). First, extract and prepare the raw data:

hcount <- rep(dist,round(dfalgae[,"Closterium_spp"]))

Then, create a randomly jittered version:

```
set.seed(123)
hcount_jittered <- rep(NA,length(hcount))
for(j in 1:length(hcount)) hcount_jittered[j] <- runif(1,min=hcount[j]-jvals[jvals$dist==hcount[j],1],
                                                       max=hcount[j]+jvals[jvals$dist==hcount[j],3])
```

Now compare histograms and kernel density estimates, using the same bins and level of smoothing, for the raw/unjittered data and the jittered version:

par(mfrow=c(2,2))
hist(hcount,breaks=50,freq=FALSE,main="Raw/unjittered Closterium_spp",xlab="Distance")
hist(hcount_jittered,breaks=50,freq=FALSE,main="Jittered Closterium_spp",xlab="Distance")
xv <- seq(0,60,length=256)
xlen <- length(xv)
fhat <- dbckden(xv,hcount,bw=1.5,bcmethod="reflect")
fjit <- dbckden(xv,hcount_jittered,bw=1.5,bcmethod="reflect")
plot(xv,fhat,type="l",xlim=range(xv),xaxs="i",main="",xlab="Distance",lwd=1.5)
abline(h=0,col="darkgrey")
plot(xv,fjit,type="l",xlim=range(xv),xaxs="i",main="",xlab="Distance",lwd=1.5)
abline(h=0,col="darkgrey")

The spurious ‘bumps’ in the distribution of the distance values are especially visible in the right tail for the unjittered version of the data; these are an artefact of the duplicated observations and are unlikely to be reflective of the true shape of the dispersion density. Note this undesirable effect is not present in the jittered version.

### Implementation of KDE and finding confidence ‘extremes’

We can use the aforementioned approach to finding confidence bands for the jittered-data density estimate. However, jittering is a random process: a different instance of the jittered data will therefore produce different confidence bands (even if only slightly). To faithfully represent the additional uncertainty in the resulting confidence bands due to the jittering, our approach in the current paper is to simulate N different randomly jittered versions of the data for a given taxon, estimating the density and finding the bootstrap confidence intervals for each. The final confidence band is taken to be the mean limits arising from each of the N versions. We also make a slight modification to the bootstrap procedure for these data, resampling only 20 data points with replacement from a given jittered data set (note size=length(dist) in the code below). This is because the jittered observations are essentially artificial, and choosing the number of observations to resample to be equal to the (much smaller) number of unique distance positions is thus a pragmatic decision made to mitigate the risk of over-emphasizing the level of precision in our estimates. In summary, the net effect is to produce confidence intervals for a given density estimate that are purposely conservative in terms of our ability to make inferences, reflecting the randomness associated with the jittering step itself.

The following code demonstrates the approach taken to generate the results in the paper. For illustrative purposes, we have simply set the number of different jitter-simulated datasets to be 2; note the N <- 2 below (the results in the paper are based on 100 simulated datasets). This is done for ease of compilation of this document; even with N <- 2 you can expect to wait several minutes for the code here to complete execution, depending on your value of PARA and the speed of your CPU cores. In addition to calculating the N density estimates via dbckden (and the bootstrap confidence bands with kdeboot) we also make use of qbckden to find a number of estimated quantile values for the purposes of summarizing the final density estimate.

```
N <- 2    # SET THE NUMBER OF JITTERED DATA SETS HERE
result <- list()
for(i in 1:N){
  hcount_jittered <- rep(NA,length(hcount))
  for(j in 1:length(hcount)) hcount_jittered[j] <- runif(1,min=hcount[j]-jvals[jvals$dist==hcount[j],1],max=hcount[j]+jvals[jvals$dist==hcount[j],3])

  h <- suppressWarnings(bw.bcv(hcount_jittered))
  f <- dbckden(xv,hcount_jittered,bw=h,bcmethod="reflect")
  qf <- qbckden(c(0.01,0.025,0.05,0.1,0.25,0.5,0.75,0.9,0.95,0.975,0.99),hcount_jittered,bw=h,bcmethod="reflect")
  fb <- kdeboot(xv,hcount_jittered,h,para=PARA,K=100,size=length(dist))

  result[[i]] <- cbind(fb[1,],c(f,qf),fb[2,])
}
result <- array(unlist(result),c(dim(result[[1]]),N))
```

[[Approx. execution time for preceding code chunk: 2.25 minutes]].

The object result produced above contains the density estimates for each of the N iterations, their corresponding 95% confidence bands, and the specified quantile values. We extract these as follows, setting the final density curve to be the average of the N density estimates, the final quantile estimates to the average of the N sets of density-based quantiles the final 95% confidence band limits to be the average of the N sets of 95% bands:

resultstats <- cbind(apply(result,1:2,mean)[,1],apply(result,1:2,mean)[,2],apply(result,1:2,mean)[,3])
y <- as.data.frame(cbind(xv,resultstats[1:xlen,]))
colnames(y) <- c("Distance","95%.Low","Mean","95%.Up")
quan <- as.data.frame(resultstats[(xlen+1):(xlen+11),])
dimnames(quan) <- list(c("Q.01","Q.025","Q.05","Q.10","Q.25","Q.50","Q.75","Q.90","Q.95","Q.975","Q.99"),c("95%.Low","Mean","95%.Up"))

Finally, using the running example of the "Closterium_spp" data, we can plot the dispersion density estimate found, complete with averaged bootstrap confidence intervals and selected quantile estimates:

```
d <- y$Distance
v <- y[,c("95%.Low","Mean","95%.Up")]
q <- round(quan,2)
## Basic plot
plot(d,v$Mean,main="Closterium_spp",type="l",ylab="Density",xlab="Distance",ylim=range(v),xaxs="i",yaxs="i")  # set up plot, labels, limits
polygon(c(d,rev(d)),c(v$`95%.Low`,rev(v$`95%.Up`)),col='gray',density=-0.5,border=FALSE) # plot confidence band
lines(d,v$Mean,col="black",lty=1,lwd=2) # plot mean density estimate on top of confidence bands
## Optional: add quantiles to plot
sel1 <- "Q.10"  # identify which quantiles (if any) you want to show on plot
sel2 <- "Q.50"
sel3 <- "Q.90"
id1 <- row.names(q)==sel1
id2 <- row.names(q)==sel2
id3 <- row.names(q)==sel3
abline(v=q[id1,2],col="yellow3",lty=2,lwd=1.5)
abline(v=q[id2,2],col="orange",lty=2,lwd=1.5)
abline(v=q[id3,2],col="red",lty=2,lwd=1.5)
legend("topright",lty=c(1,1,2,2,2),lwd=c(2,8,1.5,1.5,1.5),col=c("black","grey","yellow3","orange","red"),
       legend=c("KDE mean","95% env.",
                paste0(sel1,": ",q[id1,2]," (",q[id1,1],", ",q[id1,3],")"),
                paste0(sel2,": ",q[id2,2]," (",q[id2,1],", ",q[id2,3],")"),
                paste0(sel3,": ",q[id3,2]," (",q[id3,1],", ",q[id3,3],")")))
```

## Settling-velocity experiment data

Turning to the dispersal distances simulated from estimated settling velocities, we can take much the same approach to estimate the dispersal densities as described in the preceding section. Exact distances are observed, so the situation is simpler in terms of the computational tasks: the need to define jittering zones or generate multiple jittered versions of the data when implementing KDE (and the associated bootstrap procedure) is no longer a pressing one.

First, read in the data and define some constants:

dist_trips <- read.csv("dist_trips.csv")
dnames <- names(dist_trips)
dlen <- length(dist_trips)

There are 58 different variables for which we wish to compute density estimates. As previously, we will simply choose one, "Ankinodestrum_spp.Lake.Taxon", to illustrate the methods here. As there is no jittering, the bootstrap procedure carried out by kdeboot will use a resample size equal to the number of observations (in this case, for each of the 58 variants, there are 1000 data points).

ssdata <- dist_trips[,"Ankinodestrum_spp.Lake.Taxon"]
ssrange <- range(ssdata)
xv <- seq(0,ssrange[2],length=256)
xlen <- length(xv)
h <- bw.bcv(ssdata)
f <- dbckden(xv,ssdata,bw=h,bcmethod="reflect")
qf <- qbckden(c(0.01,0.025,0.05,0.1,0.25,0.5,0.75,0.9,0.95,0.975,0.99),ssdata,bw=h,bcmethod="reflect")
fb <- kdeboot(xv,ssdata,h,para=PARA,K=100)

[[Approx. execution time for preceding code chunk: 1.28 minutes]].

As earlier, we can extract the density estimate, bootstrap confidence bands, and desired quantile values, and plot the result:

```
plot(xv,f,main="Ankinodestrum_spp.Lake.Taxon",type="l",ylab="Density",xlab="Distance",ylim=range(fb[,1:xlen]),xaxs="i",yaxs="r",xlim=c(0,qf[9]))
polygon(c(xv,rev(xv)),c(fb[1,1:xlen],fb[2,xlen:1]),col='gray',density=-0.5,border=FALSE) # plot confidence band
lines(xv,f,col="black",lty=1,lwd=2)
abline(v=qf[c(4,6,8)],col=c("yellow3","orange","red"),lty=2,lwd=1.5)
legend("topright",lty=c(1,1,2,2,2),lwd=c(2,8,1.5,1.5,1.5),col=c("black","grey","yellow3","orange","red"),
       legend=c("KDE mean","95% env.",
                paste0("Q.10: ",round(qf[4],2)," (",round(fb[1,xlen+4],2),", ",round(fb[2,xlen+4],2),")"),
                paste0("Q.50: ",round(qf[6],2)," (",round(fb[1,xlen+6],2),", ",round(fb[2,xlen+6],2),")"),
                paste0("Q.90: ",round(qf[8],2)," (",round(fb[1,xlen+8],2),", ",round(fb[2,xlen+8],2),")")))
```

# Figure S1

**Figure S1.** Schematic overview of the settling experiment. A – Thalheim settling columns are acrylic columns with a glass chamber at the bottom that can be removed and observed under an inverted microscope. In a Thalheim settling column almost full of distilled water, we added an algal sample of 2.5 mL with a pipette and then closed the system with a plastic cover. After 5 min, we removed the chamber from the bottom and analyzed the sample under the microscope. B – For stream periphyton we used three different columns, small (18 mm), medium (51 mm) and large (106 mm). All columns had an inner diameter of 21 mm. C – To concentrate lake algal samples, we used two Thalheim columns (a large one stacked on top of a small one) and added 58 mL of lake water. After 24 hours, we removed the small column with 8 mL of concentrated sample. Each stream periphyton sample (which contained many cells) had to be diluted to allow a precise characterization of algal settling velocities, with dilution factors ranging from x2 to x6. By contrast, lake algal samples had to be concentrated, which we achieved by employing two Thalheim columns (the large one stacked on top of the small one). We first added 58 mL of lake sample to this stacked column, let it settle for 24 hours, and then removed the small column from the bottom, thus concentrating algae in just 8 mL of sample. Regrettably, the first trial of the settling experiment with lake water samples from spring was unsuccessful and had to be discarded. Thus, for phytoplankton we just used the winter lake water samples.

# Figure S2

## Figure S2a

## Figure S2b

**Figure S2.** Settled cell proportions according to column height for total cell density for periphyton samples (Figure S2a) and phytoplankton samples (Figure S2b). Empty dots represent column replicate density values, and solid dots represent means per column height. Curves were fitted using a log-normal distribution. The number of settled cells decreases according to the height of each Thalheim column, tending towards zero, thus allowing us to calculate a settling velocity. Initially, several distributions were applied to our data (log-normal, normal, gamma, chi-square and exponential), fitting parameters using the function *stats::nlm* with a Newton-type algorithm (Dennis and Schnabel 1996). The log-normal distribution resulted in the best fit (lowest f value) in 84% of all cases and the second-best fit in 7% of cases. Therefore, we applied it to all settling models for consistency (Table S5). We considered only taxa with an abundance of > 5% of the total cell count in the reference sample, thus excluding rarer taxa. Further, we examined solely taxa that were present in at least 2 column-length trials and yielded at least 8 data points for fitting the distribution. Finally, we only included results for taxa that showed significant relationships between log-transformed settled cell proportion and column length using a linear model. After applying these criteria, we ended up successfully fitting settling velocity functions for 26 algal taxa.

# Table S1

**Table S1.** The five most abundant phytoplankton taxa in each individual sample and their relative abundances (in %). Shown are phytoplankton samples taken from Sullivan Dam reservoir (Lake) during spring and winter, and from the riverbed of the Water of Leith (River) during spring and winter at different distances (in m) downstream of the Sullivan Dam outlet.

| Sample name | First taxon | First % | Second taxon | Second % | Third taxon | Third % | Fourth taxon | Fourth % | Fifth taxon | Fifth % |
| --- | --- | --- | --- | --- | --- | --- | --- | --- | --- | --- |
| Lake spring 1 | *Leptolyngbya* spp. | 42.29 | *Chlorella* spp. | 11.17 | *Euglena* spp. | 7.71 | *Monoraphidium* spp. | 6.91 | Flagellate spp. | 6.38 |
| Lake spring 2 | *Leptolyngbya* spp. | 22.55 | *Chlorella* spp. | *17.39* | *Monoraphidium* spp. | 6.79 | *Euglena* spp. | 5.98 | *Flagellate* spp. | 5.71 |
| Lake winter 1 | *Leptolyngbya* spp. | 23.64 | *Chlorella* spp. | 16.03 | *Stichococcus* spp. | 8.42 | *Euglena* spp. | 7.61 | *Peridinium* spp. | 7.61 |
| Lake winter 2 | *Chlorella* spp. | 52.29 | *Rhodomonas* spp. | 6.56 | *Botryococcus* spp. | 5.96 | *Golenkinia* spp. | 5.17 | *Flagellate* spp. | 4.97 |
| Lake winter 3 | *Chlorella* spp. | 49.22 | *Golenkinia* spp. | 7.65 | *Botryococcus* spp. | 7.45 | *Rhodomonas* spp. | 5.1 | *Flagellate* spp. | 4.12 |
| Lake winter 4 | *Chlorella* spp. | 46.91 | *Botryococcus* spp. | 8.85 | *Rhodomonas* spp. | 6.17 | *Flagellate* spp. | 5.76 | *Cyclotella* spp. | 4.12 |
| River spring 850m | *Chlorella* spp. | 13.18 | *Botryococcus* spp. | 11.63 | *Audouinella* spp. | 9.69 | *Gomphonema* spp. | 9.69 | *Lyngbya* spp. | 7.36 |
| River spring 850m | *Chlorella* spp. | 13.02 | *Oscilatoria* spp. | 13.02 | *Lyngbya* spp. | 9.9 | *Stichococcus* spp. | 9.37 | *Botryococcus* spp. | 7.29 |
| River spring 350m | *Audouinella* spp. | 23.38 | *Chlorella* spp. | 20.78 | *Botryococcus* spp. | 11.26 | *Cyclotella* spp. | 7.79 | *Monoraphidium* spp. | 5.63 |
| River spring 850m | *Chlorella* spp. | 22.55 | *Botryococcus* spp. | 17.09 | *Cyclotella* spp. | 9.82 | *Stichococcus* spp. | 7.27 | *Flagellate* spp. | 4.36 |
| River spring 350m | *Chlorella* spp. | 22.26 | *Botryococcus* spp. | 18.61 | *Monoraphidium* spp. | 6.93 | *Synedra* spp. | 6.57 | *Stichococcus* spp. | 5.47 |
| River spring 350m | *Chlorella* spp. | 28.81 | *Botryococcus* spp. | 25.85 | *Cyclotella* spp. | 5.93 | *Monoraphidium* spp. | 5.93 | *Flagellate* spp. | 3.81 |
| River spring 850m | *Botryococcus* spp. | 22.22 | *Chlorella* spp. | 21.46 | *Fragilaria* spp. | 8.05 | *Monoraphidium* spp. | 5.36 | *Synedra* spp. | 5.36 |
| River spring 850m | *Botryococcus* spp. | 22.62 | *Chlorella* spp. | 19.84 | *Monoraphidium* spp. | 5.56 | *Cyclotella* spp. | 5.16 | *Stichococcus* spp. | 4.76 |
| River spring 350m | *Chlorella* spp. | 24.01 | *Audouinella* spp. | 15.77 | *Botryococcus* spp. | 9.32 | *Lyngbya* spp. | 6.45 | *Cyclotella* spp. | 5.38 |
| River spring 350m | *Chlorella* spp. | 22.92 | *Audouinella* spp. | 17.92 | *Botryococcus* spp. | 17.08 | *Lyngbya* spp. | 5 | *Monoraphidium* spp. | 5 |
| River winter 1950m | *Tetraedron* spp. | 24.13 | *Botryococcus* spp. | 20.93 | *Chlorella* spp. | 16.57 | *Monoraphidium* spp. | 13.08 | *Eunotia* spp. | 6.1 |
| River winter 1950m | *Botryococcus* spp. | 23.87 | *Chlorella* spp. | 18.11 | *Monoraphidium* spp. | 17.28 | *Tetraedron* spp. | 13.99 | *Ankinodestrum* spp. | 4.94 |
| River winter 1950m | *Tetraedron* spp. | 29.4 | *Botryococcus* spp. | 27.14 | *Monoraphidium* spp. | 10.3 | *Chlorella* spp. | 8.29 | *Eunotia* spp. | 7.79 |
| River winter 1950m | *Botryococcus* spp. | 22.41 | *Tetraedron* spp. | 18.73 | *Monoraphidium* spp. | 14.05 | *Eunotia* spp. | 10.37 | *Chlorella* spp. | 8.03 |
| River winter 7700m | *Monoraphidium* spp. | 19.38 | *Chlorella* spp. | 10.13 | *Melosira* spp. | 9.69 | *Tetraedron* spp. | 7.93 | *Botryococcus* spp. | 7.49 |
| River winter 7700m | *Monoraphidium* spp. | 12.22 | *Chlorella* spp. | 10.86 | *Botryococcus* spp. | 10.41 | *Tetraedron* spp. | 7.69 | *Eunotia* spp. | 6.33 |
| River winter 7700m | *Botryococcus* spp. | 18.6 | *Chlorella* spp. | 14.46 | *Monoraphidium* spp. | 14.46 | *Tetraedron* spp. | 14.05 | *Flagellate* spp. | 4.96 |
| River winter 1950m | *Botryococcus* spp. | 25.47 | *Chlorella* spp. | 20.13 | *Tetraedron* spp. | 12.58 | *Eunotia* spp. | 10.69 | *Monoraphidium* spp. | 9.12 |
| River winter 7700m | *Chlorella* spp. | 19.14 | *Monoraphidium* spp. | 13.67 | *Botryococcus* spp. | 12.5 | *Eunotia* spp. | 10.16 | *Rhoicosphenia* spp. | 8.59 |
| River winter 7700m | *Rhoicosphenia* spp. | 19.74 | *Chlorella* spp. | 17.76 | *Tetraedron* spp. | 7.89 | *Monoraphidium* spp. | 6.58 | *Botryococcus* spp. | 6.25 |

# Table S2

**Table S2.** Summary results for zero-inflation models evaluating the relationships between microhabitat conditions (depth and flow velocity, Flow vel) and cell density estimated from exposed tiles. Each model summary includes results for the Count component (model with non-zero values) and the ZI component (Zero-Inflation, logistic model).

| Taxa | Term | Model | Estimate | Std_Error | Z_value | P_value |
| --- | --- | --- | --- | --- | --- | --- |
| *Closterium* spp. | Flow vel | Count | -0.918 | 0.644 | -1.425 | 0.154 |
| *Closterium* spp. | Depth | Count | 0.549 | 0.661 | 0.831 | 0.406 |
| *Closterium* spp. | Flow vel | ZI | -0.440 | 0.296 | -1.483 | 0.138 |
| *Closterium* spp. | Depth | ZI | 0.204 | 0.291 | 0.700 | 0.484 |
| *Euglena* spp. | Flow vel | Count | -0.729 | 0.948 | -0.769 | 0.442 |
| *Euglena* spp. | Depth | Count | 0.922 | 0.945 | 0.976 | 0.329 |
| *Euglena* spp. | Flow vel | ZI | -0.888 | 0.741 | -1.198 | 0.231 |
| *Euglena* spp. | Depth | ZI | -0.537 | 0.570 | -0.942 | 0.346 |
| *Dictyosphaerium* spp. | Flow vel | Count | -1.685 | 1.315 | -1.281 | 0.200 |
| *Dictyosphaerium* spp. | Depth | Count | -1.034 | 1.416 | -0.730 | 0.465 |
| *Dictyosphaerium* spp. | Flow vel | ZI | -0.509 | 0.575 | -0.886 | 0.376 |
| *Dictyosphaerium* spp. | Depth | ZI | -0.449 | 0.588 | -0.764 | 0.445 |
| *Peridinium* spp. | Flow vel | Count | -0.479 | 1.302 | -0.367 | 0.713 |
| *Peridinium* spp. | Depth | Count | 0.909 | 1.215 | 0.748 | 0.454 |
| *Peridinium* spp. | Flow vel | ZI | 0.514 | 0.334 | 1.538 | 0.124 |
| *Peridinium* spp. | Depth | ZI | 0.110 | 0.321 | 0.343 | 0.731 |
| *Cryptomonas* spp. | Flow vel | Count | -1.576 | 1.476 | -1.067 | 0.286 |
| *Cryptomonas* spp. | Depth | Count | 0.751 | 1.362 | 0.552 | 0.581 |
| *Cryptomonas* spp. | Flow vel | ZI | -0.013 | 0.402 | -0.033 | 0.974 |
| *Cryptomonas* spp. | Depth | ZI | -0.084 | 0.372 | -0.227 | 0.821 |
| *Phacus* spp. | Flow vel | Count | -0.834 | 1.134 | -0.735 | 0.462 |
| *Phacus* spp. | Depth | Count | 1.174 | 1.008 | 1.166 | 0.244 |
| *Phacus* spp. | Flow vel | ZI | -0.315 | 0.291 | -1.084 | 0.278 |
| *Phacus* spp. | Depth | ZI | 0.160 | 0.283 | 0.566 | 0.572 |
| ***Chlamydomonas* spp.** | **Flow vel** | **Count** | **-3.014** | **1.224** | **-2.462** | **0.014** |
| *Chlamydomonas* spp. | Depth | Count | -1.800 | 1.550 | -1.162 | 0.245 |
| *Chlamydomonas* spp. | Flow vel | ZI | 0.129 | 0.320 | 0.403 | 0.687 |
| *Chlamydomonas* spp. | Depth | ZI | -0.032 | 0.318 | -0.102 | 0.919 |
| Total cell density | Flow vel | Count | -0.079 | 0.044 | -1.782 | 0.075 |
| Total cell density | Depth | Count | 0.029 | 0.044 | 0.663 | 0.507 |
| All selected taxa | Flow vel | Count | -0.078 | 0.049 | -1.595 | 0.111 |
| All selected taxa | Depth | Count | -0.015 | 0.052 | -0.280 | 0.780 |

# Table S3

**Table S3.** Detailed results of fitting log-normal distributions to the settling experiment results for all taxa combined, taxonomical/morphological algal classes, and densities of 15 plankton taxa and 21 periphyton taxa. From = data origin (lake phytoplankton or river periphyton), N sample = Number of data used to fit model. P value is the output from the fitted model. Set vel med (Median), Set vel 1q (quartile), Set vel 3q (quartile), Set vel 10 (Percentile) and Set vel 90 (Percentile) are settling velocities estimated using the fitted lognormal distributions. Dist med, Dist 1q, Dist 3q, Dist 10 and Dist 90 (median distance, 1^st^ quantile, 3^rd^ quantile, percentile 10 and percentile 90) are estimated algal cell travel distances using the hydraulic model.

| Morpho class | Group | Taxon | From | N sample | P value | Set vel med | Set vel 1q | Set vel 3q | Set vel 10 | Set vel 90 | Dist med | Dist 1q | Dist 3q | Dist 90 | Dist 10 |
| --- | --- | --- | --- | --- | --- | --- | --- | --- | --- | --- | --- | --- | --- | --- | --- |
| All.taxa |  | All taxa combined | River | 36 | <0.001 | 4.99.E-03 | 1.12.E-03 | 2.15.E-02 | 1.91.E-04 | 7.95.E-02 | 0.472 | 0.169 | 1.021 | 2.060 | 0.062 |
| All.taxa |  | All taxa combined | Lake | 16 | <0.001 | 3.52.E-03 | 8.79.E-04 | 1.26.E-02 | 1.89.E-04 | 3.92.E-02 | 0.392 | 0.146 | 0.916 | 1.600 | 0.053 |
| Colonial | Morpho type | Colonial | Lake | 16 | <0.001 | 7.37.E-03 | 2.30.E-03 | 2.23.E-02 | 7.83.E-04 | 6.35.E-02 | 0.406 | 0.162 | 0.945 | 1.714 | 0.059 |
| Colonial |  | Colonial | River | 36 | <0.001 | 7.98.E-03 | 1.62.E-03 | 3.83.E-02 | 2.95.E-04 | 1.67.E-01 | 0.432 | 0.170 | 1.017 | 2.162 | 0.061 |
| Cyano. |  | Cyanobacteria | Lake | 16 | 0.16 |  |  |  |  |  |  |  |  |  |  |
| Cyano. |  | Cyanobacteria | River | 28 | 0.17 |  |  |  |  |  |  |  |  |  |  |
| Diatom |  | Diatoms | Lake | 16 | 0.01 | 2.36.E-03 | 5.08.E-04 | 9.14.E-03 | 4.02.E-05 | 3.41.E-02 | 0.390 | 0.163 | 0.837 | 1.457 | 0.061 |
| Diatom |  | Diatoms | River | 36 | <0.001 | 6.02.E-03 | 2.19.E-03 | 1.64.E-02 | 8.36.E-04 | 4.14.E-02 | 0.452 | 0.175 | 1.043 | 2.169 | 0.064 |
| Filamentous |  | Filamentous | Lake | 16 | 0.3 |  |  |  |  |  |  |  |  |  |  |
| Filamentous |  | Filamentous | River | 36 | <0.001 | 9.53.E-03 | 1.49.E-03 | 5.62.E-02 | 1.63.E-04 | 3.04.E-01 | 0.354 | 0.132 | 0.839 | 1.797 | 0.048 |
| Flagellate |  | Flagellates | River | 36 | 0.02 | 2.16.E-02 | 3.72.E-03 | 1.17.E-01 | 7.57.E-04 | 5.65.E-01 | 0.332 | 0.128 | 0.766 | 1.338 | 0.048 |
| Flagellate |  | Flagellates | Lake | 16 | <0.001 | 1.53.E-03 | 5.84.E-04 | 3.72.E-03 | 1.99.E-04 | 8.25.E-03 | 0.480 | 0.170 | 1.109 | 2.487 | 0.061 |
| S. cells |  | Single cells | Lake | 16 | <0.001 | 5.69.E-03 | 1.72.E-03 | 1.70.E-02 | 5.41.E-04 | 4.98.E-02 | 0.418 | 0.172 | 0.950 | 1.983 | 0.063 |
| S. cells |  | Single cells | River | 36 | <0.001 | 1.58.E-03 | 5.19.E-04 | 4.18.E-03 | 1.36.E-04 | 1.03.E-02 | 0.442 | 0.182 | 0.912 | 1.894 | 0.068 |
| Colonial | Taxon | *Ankinodestrum* spp. | River | 16 | 0.01 | 1.42.E-03 | 7.32.E-04 | 2.63.E-03 | 3.52.E-04 | 4.68.E-03 | 0.438 | 0.166 | 0.963 | 1.817 | 0.060 |
| Colonial |  | *Dictyosphaerium* spp. | River | 12 | 0.03 | 1.94.E-03 | 4.19.E-04 | 7.03.E-03 | 4.26.E-05 | 2.24.E-02 | 0.424 | 0.177 | 0.960 | 1.934 | 0.067 |
| Colonial |  | *Ankinodestrum* spp. | Lake | 16 | 0.04 | 1.81.E-02 | 3.70.E-03 | 8.79.E-02 | 7.69.E-04 | 3.85.E-01 | 0.397 | 0.134 | 0.983 | 1.922 | 0.047 |
| Colonial |  | *Botryococcus* spp. | Lake | 16 | <0.001 | 8.77.E-03 | 2.95.E-03 | 2.39.E-02 | 1.13.E-03 | 5.91.E-02 | 0.451 | 0.166 | 0.935 | 1.977 | 0.058 |
| Colonial |  | *Botryococcus* spp. | River | 36 | <0.001 | 1.38.E-02 | 3.05.E-03 | 5.94.E-02 | 6.90.E-04 | 2.55.E-01 | 0.330 | 0.130 | 0.808 | 1.601 | 0.046 |
| Colonial |  | *Dictyosphaerium* spp. | Lake | 16 | <0.001 | 6.57.E-03 | 2.67.E-03 | 1.59.E-02 | 1.08.E-03 | 3.73.E-02 | 0.426 | 0.159 | 0.943 | 1.920 | 0.058 |
| Cyano. |  | *Stichococcus* spp. | Lake | 16 | 0.16 |  |  |  |  |  |  |  |  |  |  |
| Cyano. |  | *Stichococcus* spp. | River | 28 | 0.17 |  |  |  |  |  |  |  |  |  |  |
| Diatom |  | *Cyclotella* spp. | Lake | 16 | 0.01 | 1.70.E-03 | 5.20.E-04 | 4.93.E-03 | 1.03.E-04 | 1.28.E-02 | 0.411 | 0.156 | 0.937 | 1.611 | 0.057 |
| Diatom |  | *Fragilaria* spp. | River | 12 | 0.01 | 1.61.E-02 | 8.98.E-03 | 2.84.E-02 | 5.29.E-03 | 4.84.E-02 | 0.448 | 0.189 | 0.986 | 1.951 | 0.069 |
| Diatom |  | *Surirella* spp. | River | 8 | 0.01 | 1.12.E-02 | 4.58.E-03 | 2.64.E-02 | 2.05.E-03 | 5.96.E-02 | 0.435 | 0.185 | 0.979 | 1.911 | 0.070 |
| Diatom |  | *Synedra* spp. | River | 12 | 0.02 | 1.45.E-03 | 5.98.E-04 | 3.15.E-03 | 2.21.E-04 | 6.59.E-03 | 0.458 | 0.175 | 1.070 | 2.001 | 0.064 |
| Diatom |  | *Rossithidium* spp. | River | 8 | 0.04 | 4.25.E-03 | 9.33.E-04 | 1.73.E-02 | 1.40.E-04 | 6.63.E-02 | 0.405 | 0.164 | 0.894 | 1.763 | 0.059 |
| Diatom |  | *Planothidium* spp. | River | 8 | 0.05 | 1.53.E-03 | 6.46.E-04 | 3.25.E-03 | 2.62.E-04 | 6.61.E-03 | 0.465 | 0.183 | 0.961 | 1.972 | 0.067 |
| Diatom |  | *Nitzschia* spp. | River | 24 | 0.09 |  |  |  |  |  |  |  |  |  |  |
| Diatom |  | *Frustulia* spp. | River | 12 | 0.33 |  |  |  |  |  |  |  |  |  |  |
| Diatom |  | *Cyclotella* spp. | River | 20 | <0.001 | 1.46.E-03 | 6.82.E-04 | 2.94.E-03 | 3.11.E-04 | 5.58.E-03 | 0.420 | 0.176 | 0.966 | 1.782 | 0.065 |
| Diatom |  | *Achnanthidium* spp. | River | 36 | <0.001 | 1.54.E-02 | 9.10.E-03 | 2.60.E-02 | 5.73.E-03 | 4.32.E-02 | 0.481 | 0.190 | 1.012 | 2.058 | 0.068 |
| Diatom |  | *Cocconeis* spp. | River | 28 | <0.001 | 8.25.E-03 | 3.26.E-03 | 2.05.E-02 | 1.36.E-03 | 4.91.E-02 | 0.418 | 0.169 | 0.942 | 1.744 | 0.063 |
| Diatom |  | *Gomphonema* spp. | River | 28 | <0.001 | 1.86.E-02 | 1.06.E-02 | 3.20.E-02 | 6.28.E-03 | 5.42.E-02 | 0.430 | 0.173 | 0.950 | 1.810 | 0.063 |
| Diatom |  | *Navicula* spp. | River | 24 | <0.001 | 1.26.E-02 | 4.45.E-03 | 3.65.E-02 | 1.67.E-03 | 1.00.E-01 | 0.444 | 0.152 | 0.918 | 1.689 | 0.055 |
| Filamentous |  | *Oscilatoria* spp. | River | 32 | 0.02 | 3.31.E-02 | 1.01.E-02 | 1.03.E-01 | 3.60.E-03 | 2.92.E-01 | 0.368 | 0.123 | 0.856 | 1.744 | 0.042 |
| Filamentous |  | *Lyngbya* spp. | River | 12 | 0.03 | 9.40.E-04 | 6.44.E-04 | 1.34.E-03 | 4.48.E-04 | 1.83.E-03 | 0.451 | 0.181 | 1.039 | 2.170 | 0.066 |
| Filamentous |  | *Leptolyngbya* spp. | Lake | 12 | 0.36 |  |  |  |  |  |  |  |  |  |  |
| Filamentous |  | *Audouinella* spp. | River | 28 | 0.59 |  |  |  |  |  |  |  |  |  |  |
| Filamentous |  | *Leptolyngbya* spp. | River | 16 | <0.001 | 1.25.E-03 | 6.16.E-04 | 2.42.E-03 | 3.02.E-04 | 4.30.E-03 | 0.417 | 0.163 | 0.913 | 1.707 | 0.060 |
| Flagellate |  | *Rhodomonas* spp. | Lake | 12 | 0.03 | 9.46.E-04 | 6.43.E-04 | 1.33.E-03 | 4.48.E-04 | 1.83.E-03 | 0.505 | 0.184 | 1.128 | 2.200 | 0.067 |
| Flagellate |  | *Phacus* spp. | River | 12 | 0.04 | 3.56.E-03 | 6.95.E-04 | 1.55.E-02 | 6.82.E-05 | 6.21.E-02 | 0.379 | 0.142 | 0.864 | 1.789 | 0.050 |
| Flagellate |  | *Cryptomonas* spp. | River | 8 | 0.16 |  |  |  |  |  |  |  |  |  |  |
| Flagellate |  | *Euglena* spp. | River | 20 | 0.18 |  |  |  |  |  |  |  |  |  |  |
| Flagellate |  | *Flagellate* spp. | River | 20 | 0.3 |  |  |  |  |  |  |  |  |  |  |
| Flagellate |  | *Euglena* spp. | Lake | 16 | 0.76 |  |  |  |  |  |  |  |  |  |  |
| Flagellate |  | *Phacus* spp. | Lake | 8 | 0.94 |  |  |  |  |  |  |  |  |  |  |
| Flagellate |  | *Trachelomonas* spp. | River | 20 | 0.99 |  |  |  |  |  |  |  |  |  |  |
| Flagellate |  | *Flagellate* spp. | Lake | 16 | <0.001 | 2.63.E-03 | 7.57.E-04 | 8.54.E-03 | 1.60.E-04 | 2.53.E-02 | 0.460 | 0.180 | 1.126 | 2.246 | 0.066 |
| Flagellate |  | *Peridinium* spp. | River | 24 | <0.001 | 1.18.E-02 | 5.62.E-03 | 2.43.E-02 | 2.89.E-03 | 4.89.E-02 | 0.435 | 0.172 | 0.997 | 1.942 | 0.063 |
| S. cells |  | *Closterium* spp. | River | 16 | 0.01 | 4.23.E-03 | 1.30.E-03 | 1.26.E-02 | 4.10.E-04 | 3.49.E-02 | 0.439 | 0.178 | 0.921 | 1.754 | 0.067 |
| S. cells |  | *Monoraphidium* spp. | Lake | 16 | 0.01 | 3.29.E-02 | 3.03.E-02 | 3.60.E-02 | 2.80.E-02 | 3.88.E-02 | 0.421 | 0.182 | 0.915 | 1.609 | 0.071 |
| S. cells |  | *Selenastrum* spp. | Lake | 12 | 0.1 |  |  |  |  |  |  |  |  |  |  |
| S. cells |  | *Golenkinia* spp. | Lake | 12 | 0.14 |  |  |  |  |  |  |  |  |  |  |
| S. cells |  | *Closterium* spp. | Lake | 8 | 0.45 |  |  |  |  |  |  |  |  |  |  |
| S. cells |  | *Chlorella* spp. | River | 36 | <0.001 | 1.54.E-03 | 5.12.E-04 | 4.04.E-03 | 1.42.E-04 | 9.80.E-03 | 0.435 | 0.177 | 1.010 | 2.214 | 0.067 |
| S. cells |  | *Chlorella* spp. | Lake | 16 | <0.001 | 5.98.E-03 | 2.26.E-03 | 1.56.E-02 | 9.15.E-04 | 3.80.E-02 | 0.391 | 0.161 | 0.828 | 1.505 | 0.059 |
| S. cells |  | *Monoraphidium* spp. | River | 36 | <0.001 | 2.44.E-03 | 5.48.E-04 | 9.57.E-03 | 5.21.E-05 | 3.28.E-02 | 0.440 | 0.150 | 0.977 | 1.929 | 0.053 |

# Table S4

**Table S4.** Estimated dispersal distances obtained from the empirical dispersal kernel for selected phytoplankton taxa and all selected taxa combined. Dist med, Dist 1q, Dist 3q, Dist 10, and Dist 90 (all expressed in km) represent the median, 1^st^ quantile, 3^rd^ quantile, 10^th^ percentile and 90^th^ percentile, respectively, of each kernel density probability.

| Taxa | Dist med | Dist 1q | Dist 3q | Dist 90 | Dist 10 |
| --- | --- | --- | --- | --- | --- |
| *Closterium* spp. | 0.437 | 0.190 | 1.062 | 3.264 | 0.074 |
| *Euglena* spp. | 0.419 | 0.176 | 0.723 | 1.197 | 0.068 |
| *Dictyosphaerium* spp. | 0.431 | 0.179 | 0.760 | 1.415 | 0.070 |
| *Peridinium* spp. | 0.400 | 0.168 | 0.805 | 1.592 | 0.065 |
| *Cryptomonas* spp. | 0.299 | 0.131 | 0.632 | 1.176 | 0.051 |
| *Phacus* spp. | 0.472 | 0.189 | 0.982 | 2.075 | 0.072 |
| *Chlamydomonas* spp. | 0.586 | 0.256 | 1.288 | 4.515 | 0.099 |
| All taxa combined | 0.426 | 0.164 | 0.867 | 2.274 | 0.065 |

# Table S5

**Table S5.** Fitting adjustments for different density distributions. These distributions were fitted to the settling proportion for each taxon group as a function of settling velocity (column height/5 min). Taxa = name of taxon and taxon groups assessed. From = data origin (lake phytoplankton or stream periphyton). Distr = distribution model (norm = normal, lognorm = log-normal, gamma = gamma, chisq = chi-square, exp = exponential). f_min = optimization parameter (the lower, the better). AIC = optimization parameter corrected for the number of distribution parameters (the lower, the better). ΔAIC = difference between the lowest AIC and the rest of the AIC values for each taxon group. Δf = difference between the lowest f value and the remaining f values for each taxon group.

| Taxa | From | Distr | f_min | AIC | ΔAIC | Δf |
| --- | --- | --- | --- | --- | --- | --- |
| *Ankinodestrum* spp. | Lake | norm | 1.92 | 7.85 | 2.092 | 0.144 |
| *Ankinodestrum* spp. | Lake | lognorm | 1.78 | 7.56 | 1.803 | 0.000 |
| *Ankinodestrum* spp. | Lake | gamma | 5.29 | 14.59 | 8.834 | 3.515 |
| *Ankinodestrum* spp. | Lake | chisq | 2.77 | 7.53 | 1.780 | 0.988 |
| *Ankinodestrum* spp. | Lake | exp | 1.88 | 5.75 | 0.000 | 0.098 |
| *Botryococcus* spp. | Lake | norm | 0.37 | 4.74 | 2.333 | 0.206 |
| *Botryococcus* spp. | Lake | lognorm | 0.16 | 4.32 | 1.920 | 0.000 |
| *Botryococcus* spp. | Lake | gamma | 2.12 | 8.24 | 5.839 | 1.959 |
| *Botryococcus* spp. | Lake | chisq | 0.94 | 3.87 | 1.470 | 0.775 |
| *Botryococcus* spp. | Lake | exp | 0.20 | 2.40 | 0.000 | 0.040 |
| *Chlorella* spp. | Lake | norm | 1.18 | 6.36 | 2.344 | 0.212 |
| *Chlorella* spp. | Lake | lognorm | 0.97 | 5.93 | 1.920 | 0.000 |
| *Chlorella* spp. | Lake | gamma | 2.31 | 8.62 | 4.604 | 1.342 |
| *Chlorella* spp. | Lake | chisq | 1.95 | 5.90 | 1.891 | 0.985 |
| *Chlorella* spp. | Lake | exp | 1.01 | 4.01 | 0.000 | 0.040 |
| *Closterium* spp. | Lake | norm | 1.63 | 7.27 | 2.126 | 0.106 |
| *Closterium* spp. | Lake | lognorm | 1.53 | 7.06 | 1.914 | 0.000 |
| *Closterium* spp. | Lake | gamma | 3.82 | 11.63 | 6.492 | 2.289 |
| *Closterium* spp. | Lake | chisq | 2.06 | 6.13 | 0.984 | 0.535 |
| *Closterium* spp. | Lake | exp | 1.57 | 5.14 | 0.000 | 0.043 |
| *Cyclotella* spp. | Lake | norm | 0.30 | 4.59 | 2.138 | 0.130 |
| *Cyclotella* spp. | Lake | lognorm | 0.17 | 4.34 | 1.878 | 0.000 |
| *Cyclotella* spp. | Lake | gamma | 0.30 | 4.59 | 2.138 | 0.130 |
| *Cyclotella* spp. | Lake | chisq | 3.09 | 8.19 | 5.732 | 2.927 |
| *Cyclotella* spp. | Lake | exp | 0.23 | 2.46 | 0.000 | 0.061 |
| *Dictyosphaerium* spp. | Lake | norm | 0.83 | 5.65 | 2.319 | 0.179 |
| *Dictyosphaerium* spp. | Lake | lognorm | 0.65 | 5.29 | 1.962 | 0.000 |
| *Dictyosphaerium* spp. | Lake | gamma | 2.06 | 8.12 | 4.788 | 1.413 |
| *Dictyosphaerium* spp. | Lake | chisq | 1.57 | 5.14 | 1.804 | 0.921 |
| *Dictyosphaerium* spp. | Lake | exp | 0.67 | 3.33 | 0.000 | 0.019 |
| *Euglena* spp. | Lake | norm | 18.53 | 41.07 | 10.844 | 4.456 |
| *Euglena* spp. | Lake | lognorm | 14.08 | 32.15 | 1.933 | 0.000 |
| *Euglena* spp. | Lake | gamma | 26.96 | 57.91 | 27.689 | 12.878 |
| *Euglena* spp. | Lake | chisq | 14.11 | 30.22 | 0.000 | 0.034 |
| *Euglena* spp. | Lake | exp | 14.11 | 30.22 | < 0.001 | 0.034 |
| *Flagellate* spp. | Lake | norm | 0.34 | 4.68 | 2.094 | 0.152 |
| *Flagellate* spp. | Lake | lognorm | 0.19 | 4.37 | 1.791 | 0.000 |
| *Flagellate* spp. | Lake | gamma | 0.65 | 5.31 | 2.724 | 0.467 |
| *Flagellate* spp. | Lake | chisq | 2.27 | 6.53 | 3.949 | 2.079 |
| *Flagellate* spp. | Lake | exp | 0.29 | 2.58 | 0.000 | 0.104 |
| *Golenkinia* spp. | Lake | norm | 0.31 | 4.62 | 2.000 | 0.000 |
| *Golenkinia* spp. | Lake | lognorm | 0.31 | 4.62 | 2.000 | < 0.001 |
| *Golenkinia* spp. | Lake | gamma | 0.31 | 4.62 | 2.000 | 0.000 |
| *Golenkinia* spp. | Lake | chisq | 0.31 | 2.62 | 0.000 | < 0.001 |
| *Golenkinia* spp. | Lake | exp | 0.33 | 2.67 | 0.051 | 0.026 |
| *Leptolyngbya* spp. | Lake | norm | 3.33 | 10.66 | 2.128 | 0.163 |
| *Leptolyngbya* spp. | Lake | lognorm | 3.17 | 10.33 | 1.803 | 0.000 |
| *Leptolyngbya* spp. | Lake | gamma | 6.71 | 17.42 | 8.891 | 3.544 |
| *Leptolyngbya* spp. | Lake | chisq | 4.18 | 10.35 | 1.821 | 1.009 |
| *Leptolyngbya* spp. | Lake | exp | 3.27 | 8.53 | 0.000 | 0.099 |
| *Monoraphidium* spp. | Lake | norm | 1.79 | 7.58 | 0.957 | 0.000 |
| *Monoraphidium* spp. | Lake | lognorm | 1.79 | 7.58 | 0.957 | < 0.001 |
| *Monoraphidium* spp. | Lake | gamma | 8.86 | 21.71 | 15.092 | 7.068 |
| *Monoraphidium* spp. | Lake | chisq | 3.79 | 9.58 | 2.963 | 2.003 |
| *Monoraphidium* spp. | Lake | exp | 2.31 | 6.62 | 0.000 | 0.521 |
| *Phacus* spp. | Lake | norm | 1.13 | 6.25 | 2.001 | 0.392 |
| *Phacus* spp. | Lake | lognorm | 0.73 | 5.47 | 1.218 | 0.000 |
| *Phacus* spp. | Lake | gamma | 2.64 | 9.27 | 5.022 | 1.902 |
| *Phacus* spp. | Lake | chisq | 1.62 | 5.23 | 0.981 | 0.882 |
| *Phacus* spp. | Lake | exp | 1.12 | 4.25 | 0.000 | 0.391 |
| *Rhodomonas* spp. | Lake | norm | 0.00 | 4.00 | 2.000 | < 0.001 |
| *Rhodomonas* spp. | Lake | lognorm | 0.00 | 4.00 | 2.000 | < 0.001 |
| *Rhodomonas* spp. | Lake | gamma | 0.00 | 4.00 | 2.000 | 0.000 |
| *Rhodomonas* spp. | Lake | chisq | 0.00 | 2.00 | 0.000 | < 0.001 |
| *Rhodomonas* spp. | Lake | exp | 0.03 | 2.05 | 0.052 | 0.026 |
| *Selenastrum* spp. | Lake | norm | 13.05 | 30.10 | 9.120 | 4.422 |
| *Selenastrum* spp. | Lake | lognorm | 8.63 | 21.26 | 0.275 | 0.000 |
| *Selenastrum* spp. | Lake | gamma | 19.95 | 43.90 | 22.915 | 11.320 |
| *Selenastrum* spp. | Lake | chisq | 9.91 | 21.83 | 0.846 | 1.285 |
| *Selenastrum* spp. | Lake | exp | 9.49 | 20.98 | 0.000 | 0.862 |
| *Stichococcus* spp. | Lake | norm | 19.49 | 42.98 | 25.860 | 12.369 |
| *Stichococcus* spp. | Lake | lognorm | 7.12 | 18.24 | 1.122 | 0.000 |
| *Stichococcus* spp. | Lake | gamma | 27.72 | 59.45 | 42.326 | 20.602 |
| *Stichococcus* spp. | Lake | chisq | 7.56 | 17.12 | 0.000 | 0.439 |
| *Stichococcus* spp. | Lake | exp | 7.56 | 17.12 | < 0.001 | 0.439 |
| All taxa | Lake | norm | 0.33 | 4.66 | 2.260 | 0.275 |
| All taxa | Lake | lognorm | 0.05 | 4.11 | 1.710 | 0.000 |
| All taxa | Lake | gamma | 0.82 | 5.63 | 3.231 | 0.761 |
| All taxa | Lake | chisq | 1.78 | 5.57 | 3.169 | 1.730 |
| All taxa | Lake | exp | 0.20 | 2.40 | 0.000 | 0.145 |
| Diatoms | Lake | norm | 0.89 | 5.77 | 2.068 | 0.273 |
| Diatoms | Lake | lognorm | 0.61 | 5.23 | 1.523 | 0.000 |
| Diatoms | Lake | gamma | 1.03 | 6.05 | 2.352 | 0.414 |
| Diatoms | Lake | chisq | 3.07 | 8.14 | 4.442 | 2.459 |
| Diatoms | Lake | exp | 0.85 | 3.70 | 0.000 | 0.239 |
| Colonial | Lake | norm | 0.31 | 4.62 | 2.346 | 0.239 |
| Colonial | Lake | lognorm | 0.07 | 4.14 | 1.869 | 0.000 |
| Colonial | Lake | gamma | 1.83 | 7.65 | 5.379 | 1.755 |
| Colonial | Lake | chisq | 0.98 | 3.97 | 1.696 | 0.914 |
| Colonial | Lake | exp | 0.14 | 2.27 | 0.000 | 0.065 |
| Cyanobacteria | Lake | norm | 19.89 | 43.78 | 26.126 | 12.502 |
| Cyanobacteria | Lake | lognorm | 7.39 | 18.78 | 1.122 | 0.000 |
| Cyanobacteria | Lake | gamma | 28.13 | 60.25 | 42.592 | 20.735 |
| Cyanobacteria | Lake | chisq | 7.83 | 17.66 | 0.000 | 0.439 |
| Cyanobacteria | Lake | exp | 7.83 | 17.66 | < 0.001 | 0.439 |
| Filamentous | Lake | norm | 10.03 | 24.07 | 10.839 | 4.492 |
| Filamentous | Lake | lognorm | 5.54 | 15.08 | 1.855 | 0.000 |
| Filamentous | Lake | gamma | 16.36 | 36.73 | 23.500 | 10.822 |
| Filamentous | Lake | chisq | 5.94 | 13.88 | 0.652 | 0.399 |
| Filamentous | Lake | exp | 5.61 | 13.23 | 0.000 | 0.072 |
| Flagellates | Lake | norm | 0.06 | 4.11 | 2.000 | 0.016 |
| Flagellates | Lake | lognorm | 0.04 | 4.08 | 1.968 | 0.000 |
| Flagellates | Lake | gamma | 0.12 | 4.24 | 2.125 | 0.078 |
| Flagellates | Lake | chisq | 0.06 | 2.11 | 0.000 | 0.016 |
| Flagellates | Lake | exp | 0.07 | 2.13 | 0.018 | 0.025 |
| Single cells | Lake | norm | 1.01 | 6.03 | 2.406 | 0.287 |
| Single cells | Lake | lognorm | 0.73 | 5.45 | 1.832 | 0.000 |
| Single cells | Lake | gamma | 2.04 | 8.08 | 4.461 | 1.314 |
| Single cells | Lake | chisq | 1.86 | 5.72 | 2.097 | 1.132 |
| Single cells | Lake | exp | 0.81 | 3.62 | 0.000 | 0.084 |
| *Achnanthidium* spp. | River | norm | 12.77 | 29.53 | 2.152 | 0.309 |
| *Achnanthidium* spp. | River | lognorm | 12.46 | 28.91 | 1.533 | 0.000 |
| *Achnanthidium* spp. | River | gamma | 22.26 | 48.51 | 21.134 | 9.801 |
| *Achnanthidium* spp. | River | chisq | 12.80 | 27.60 | 0.217 | 0.342 |
| *Achnanthidium* spp. | River | exp | 12.69 | 27.38 | 0.000 | 0.234 |
| *Ankinodestrum* spp. | River | norm | 0.02 | 4.04 | 2.000 | 0.000 |
| *Ankinodestrum* spp. | River | lognorm | 0.02 | 4.04 | 1.999 | 0.000 |
| *Ankinodestrum* spp. | River | gamma | 0.03 | 4.07 | 2.027 | 0.014 |
| *Ankinodestrum* spp. | River | chisq | 0.02 | 2.04 | 0.000 | 0.000 |
| *Ankinodestrum* spp. | River | exp | 0.04 | 2.08 | 0.039 | 0.020 |
| *Audouinella* spp. | River | norm | 34.52 | 73.04 | 27.309 | 12.654 |
| *Audouinella* spp. | River | lognorm | 21.87 | 47.73 | 2.000 | < 0.001 |
| *Audouinella* spp. | River | gamma | 44.15 | 92.30 | 46.569 | 22.284 |
| *Audouinella* spp. | River | chisq | 21.87 | 45.73 | 0.000 | 0.000 |
| *Audouinella* spp. | River | exp | 21.87 | 45.73 | < 0.001 | < 0.001 |
| *Botryococcus* spp. | River | norm | 1.37 | 6.74 | 2.472 | 0.522 |
| *Botryococcus* spp. | River | lognorm | 0.85 | 5.69 | 1.428 | 0.000 |
| *Botryococcus* spp. | River | gamma | 7.73 | 19.46 | 15.198 | 6.885 |
| *Botryococcus* spp. | River | chisq | 3.09 | 8.19 | 3.920 | 2.246 |
| *Botryococcus* spp. | River | exp | 1.13 | 4.27 | 0.000 | 0.286 |
| *Chlorella* spp. | River | norm | 0.42 | 4.85 | 1.965 | 0.071 |
| *Chlorella* spp. | River | lognorm | 0.35 | 4.70 | 1.824 | 0.000 |
| *Chlorella* spp. | River | gamma | 0.56 | 5.11 | 2.232 | 0.204 |
| *Chlorella* spp. | River | chisq | 7.17 | 16.34 | 13.455 | 6.815 |
| *Chlorella* spp. | River | exp | 0.44 | 2.88 | 0.000 | 0.088 |
| *Closterium* spp. | River | norm | 1.71 | 7.43 | 2.275 | 0.211 |
| *Closterium* spp. | River | lognorm | 1.50 | 7.01 | 1.852 | 0.000 |
| *Closterium* spp. | River | gamma | 2.41 | 8.81 | 3.660 | 0.904 |
| *Closterium* spp. | River | chisq | 2.91 | 7.82 | 2.664 | 1.406 |
| *Closterium* spp. | River | exp | 1.58 | 5.15 | 0.000 | 0.074 |
| *Cocconeis* spp. | River | norm | 1.17 | 6.34 | 2.516 | 0.354 |
| *Cocconeis* spp. | River | lognorm | 0.81 | 5.63 | 1.807 | 0.000 |
| *Cocconeis* spp. | River | gamma | 4.25 | 12.49 | 8.669 | 3.431 |
| *Cocconeis* spp. | River | chisq | 1.98 | 5.96 | 2.134 | 1.163 |
| *Cocconeis* spp. | River | exp | 0.91 | 3.82 | 0.000 | 0.096 |
| *Cryptomonas* spp. | River | norm | 0.72 | 5.45 | 2.176 | 0.181 |
| *Cryptomonas* spp. | River | lognorm | 0.54 | 5.08 | 1.814 | 0.000 |
| *Cryptomonas* spp. | River | gamma | 1.52 | 7.03 | 3.763 | 0.975 |
| *Cryptomonas* spp. | River | chisq | 0.98 | 3.97 | 0.697 | 0.442 |
| *Cryptomonas* spp. | River | exp | 0.63 | 3.27 | 0.000 | 0.093 |
| *Cyclotella* spp. | River | norm | 0.14 | 4.28 | 2.000 | 0.002 |
| *Cyclotella* spp. | River | lognorm | 0.14 | 4.28 | 1.996 | 0.000 |
| *Cyclotella* spp. | River | gamma | 0.18 | 4.35 | 2.071 | 0.038 |
| *Cyclotella* spp. | River | chisq | 0.14 | 2.28 | 0.000 | 0.002 |
| *Cyclotella* spp. | River | exp | 0.16 | 2.32 | 0.039 | 0.021 |
| *Dictyosphaerium* spp. | River | norm | 0.52 | 5.05 | 2.004 | 0.120 |
| *Dictyosphaerium* spp. | River | lognorm | 0.40 | 4.81 | 1.763 | 0.000 |
| *Dictyosphaerium* spp. | River | gamma | 0.57 | 5.15 | 2.108 | 0.172 |
| *Dictyosphaerium* spp. | River | chisq | 2.48 | 6.96 | 3.923 | 2.080 |
| *Dictyosphaerium* spp. | River | exp | 0.52 | 3.04 | 0.000 | 0.118 |
| *Euglena* spp. | River | norm | 27.36 | 58.73 | 13.374 | 5.687 |
| *Euglena* spp. | River | lognorm | 21.84 | 47.67 | 2.318 | 0.159 |
| *Euglena* spp. | River | gamma | 40.17 | 84.33 | 38.979 | 18.490 |
| *Euglena* spp. | River | chisq | 21.84 | 45.67 | 0.318 | 0.159 |
| *Euglena* spp. | River | exp | 21.68 | 45.35 | 0.000 | 0.000 |
| *Flagellate* spp. | River | norm | 18.23 | 40.47 | 5.243 | 1.673 |
| *Flagellate* spp. | River | lognorm | 16.56 | 37.12 | 1.898 | 0.000 |
| *Flagellate* spp. | River | gamma | 25.18 | 54.36 | 19.132 | 8.617 |
| *Flagellate* spp. | River | chisq | 18.23 | 38.47 | 3.243 | 1.673 |
| *Flagellate* spp. | River | exp | 16.61 | 35.23 | 0.000 | 0.051 |
| *Fragilaria* spp. | River | norm | 1.79 | 7.58 | 1.883 | 0.000 |
| *Fragilaria* spp. | River | lognorm | 1.84 | 7.69 | 1.993 | 0.055 |
| *Fragilaria* spp. | River | gamma | 4.68 | 13.35 | 7.658 | 2.888 |
| *Fragilaria* spp. | River | chisq | 1.92 | 5.84 | 0.140 | 0.129 |
| *Fragilaria* spp. | River | exp | 1.85 | 5.70 | 0.000 | 0.059 |
| *Frustulia* spp. | River | norm | 14.80 | 33.59 | 14.063 | 6.056 |
| *Frustulia* spp. | River | lognorm | 8.74 | 21.48 | 1.951 | 0.000 |
| *Frustulia* spp. | River | gamma | 25.37 | 54.74 | 35.214 | 16.631 |
| *Frustulia* spp. | River | chisq | 8.76 | 19.53 | 0.000 | 0.024 |
| *Frustulia* spp. | River | exp | 8.76 | 19.53 | 0.000 | 0.024 |
| *Gomphonema* spp. | River | norm | 4.64 | 13.28 | 2.536 | 0.442 |
| *Gomphonema* spp. | River | lognorm | 4.20 | 12.40 | 1.652 | 0.000 |
| *Gomphonema* spp. | River | gamma | 13.04 | 30.07 | 19.329 | 8.839 |
| *Gomphonema* spp. | River | chisq | 4.64 | 11.28 | 0.537 | 0.442 |
| *Gomphonema* spp. | River | exp | 4.37 | 10.74 | 0.000 | 0.174 |
| *Leptolyngbya* spp. | River | norm | 0.05 | 4.10 | 2.016 | 0.009 |
| *Leptolyngbya* spp. | River | lognorm | 0.04 | 4.08 | 1.998 | 0.000 |
| *Leptolyngbya* spp. | River | gamma | 0.05 | 4.10 | 2.016 | 0.009 |
| *Leptolyngbya* spp. | River | chisq | 0.04 | 2.08 | 0.000 | 0.001 |
| *Leptolyngbya* spp. | River | exp | 0.06 | 2.13 | 0.044 | 0.023 |
| *Lyngbya* spp. | River | norm | 0.00 | 4.00 | 2.000 | < 0.001 |
| *Lyngbya* spp. | River | lognorm | 0.00 | 4.00 | 2.000 | < 0.001 |
| *Lyngbya* spp. | River | gamma | 0.00 | 4.00 | 2.000 | 0.000 |
| *Lyngbya* spp. | River | chisq | 0.00 | 2.00 | 0.000 | < 0.001 |
| *Lyngbya* spp. | River | exp | 0.03 | 2.05 | 0.052 | 0.026 |
| *Monoraphidium* spp. | River | norm | 1.63 | 7.26 | 2.142 | 0.484 |
| *Monoraphidium* spp. | River | lognorm | 1.14 | 6.29 | 1.175 | 0.000 |
| *Monoraphidium* spp. | River | gamma | 2.11 | 8.23 | 3.114 | 0.969 |
| *Monoraphidium* spp. | River | chisq | 6.53 | 15.06 | 9.950 | 5.388 |
| *Monoraphidium* spp. | River | exp | 1.56 | 5.11 | 0.000 | 0.413 |
| *Navicula* spp. | River | norm | 1.87 | 7.73 | 2.718 | 0.480 |
| *Navicula* spp. | River | lognorm | 1.39 | 6.77 | 1.759 | 0.000 |
| *Navicula* spp. | River | gamma | 6.02 | 16.04 | 11.022 | 4.631 |
| *Navicula* spp. | River | chisq | 2.33 | 6.67 | 1.651 | 0.946 |
| *Navicula* spp. | River | exp | 1.51 | 5.02 | 0.000 | 0.120 |
| *Nitzschia* spp. | River | norm | 12.31 | 28.62 | 8.504 | 3.356 |
| *Nitzschia* spp. | River | lognorm | 8.95 | 21.91 | 1.793 | 0.000 |
| *Nitzschia* spp. | River | gamma | 20.56 | 45.11 | 24.997 | 11.602 |
| *Nitzschia* spp. | River | chisq | 10.80 | 23.60 | 3.486 | 1.847 |
| *Nitzschia* spp. | River | exp | 9.06 | 20.12 | 0.000 | 0.104 |
| *Oscilatoria* spp. | River | norm | 5.38 | 14.76 | 2.540 | 0.374 |
| *Oscilatoria* spp. | River | lognorm | 5.01 | 14.02 | 1.792 | 0.000 |
| *Oscilatoria* spp. | River | gamma | 16.34 | 36.68 | 24.453 | 11.330 |
| *Oscilatoria* spp. | River | chisq | 7.29 | 16.58 | 4.359 | 2.283 |
| *Oscilatoria* spp. | River | exp | 5.11 | 12.22 | 0.000 | 0.104 |
| *Planothidium* spp. | River | norm | 0.02 | 4.04 | 2.000 | 0.003 |
| *Planothidium* spp. | River | lognorm | 0.02 | 4.03 | 1.994 | 0.000 |
| *Planothidium* spp. | River | gamma | 0.04 | 4.08 | 2.044 | 0.025 |
| *Planothidium* spp. | River | chisq | 0.02 | 2.04 | 0.000 | 0.003 |
| *Planothidium* spp. | River | exp | 0.03 | 2.05 | 0.012 | 0.009 |
| *Peridinium* spp. | River | norm | 5.35 | 14.70 | 2.319 | 0.162 |
| *Peridinium* spp. | River | lognorm | 5.19 | 14.38 | 1.996 | 0.000 |
| *Peridinium* spp. | River | gamma | 9.48 | 22.95 | 10.574 | 4.289 |
| *Peridinium* spp. | River | chisq | 5.75 | 13.50 | 1.118 | 0.561 |
| *Peridinium* spp. | River | exp | 5.19 | 12.38 | 0.000 | 0.002 |
| *Phacus* spp. | River | norm | 0.68 | 5.36 | 2.187 | 0.298 |
| *Phacus* spp. | River | lognorm | 0.38 | 4.76 | 1.591 | 0.000 |
| *Phacus* spp. | River | gamma | 1.01 | 6.02 | 2.848 | 0.628 |
| *Phacus* spp. | River | chisq | 1.84 | 5.67 | 2.501 | 1.455 |
| *Phacus* spp. | River | exp | 0.58 | 3.17 | 0.000 | 0.204 |
| *Rossithidium* spp. | River | norm | 0.40 | 4.81 | 2.263 | 0.247 |
| *Rossithidium* spp. | River | lognorm | 0.16 | 4.31 | 1.768 | 0.000 |
| *Rossithidium* spp. | River | gamma | 0.68 | 5.35 | 2.807 | 0.519 |
| *Rossithidium* spp. | River | chisq | 0.98 | 3.96 | 1.417 | 0.825 |
| *Rossithidium* spp. | River | exp | 0.27 | 2.54 | 0.000 | 0.116 |
| *Stichococcus* spp. | River | norm | 107.77 | 219.55 | 36.314 | 17.157 |
| *Stichococcus* spp. | River | lognorm | 106.29 | 216.58 | 33.348 | 15.674 |
| *Stichococcus* spp. | River | gamma | 126.45 | 256.91 | 73.674 | 35.837 |
| *Stichococcus* spp. | River | chisq | 90.62 | 183.23 | 0.000 | 0.000 |
| *Stichococcus* spp. | River | exp | 93.04 | 188.09 | 4.855 | 2.428 |
| *Surirella* spp. | River | norm | 0.18 | 4.35 | 2.199 | 0.121 |
| *Surirella* spp. | River | lognorm | 0.06 | 4.11 | 1.956 | 0.000 |
| *Surirella* spp. | River | gamma | 1.40 | 6.80 | 4.644 | 1.344 |
| *Surirella* spp. | River | chisq | 0.31 | 2.62 | 0.462 | 0.253 |
| *Surirella* spp. | River | exp | 0.08 | 2.15 | 0.000 | 0.022 |
| *Synedra* spp. | River | norm | 0.12 | 4.23 | 2.000 | 0.006 |
| *Synedra* spp. | River | lognorm | 0.11 | 4.22 | 1.987 | 0.000 |
| *Synedra* spp. | River | gamma | 0.14 | 4.29 | 2.053 | 0.033 |
| *Synedra* spp. | River | chisq | 0.12 | 2.23 | 0.000 | 0.006 |
| *Synedra* spp. | River | exp | 0.13 | 2.25 | 0.020 | 0.016 |
| *Trachelomonas* spp. | River | norm | 32.71 | 69.42 | 5.475 | 2.270 |
| *Trachelomonas* spp. | River | lognorm | 30.44 | 64.88 | 0.934 | 0.000 |
| *Trachelomonas* spp. | River | gamma | 39.45 | 82.90 | 18.951 | 9.008 |
| *Trachelomonas* spp. | River | chisq | 38.83 | 79.65 | 15.706 | 8.386 |
| *Trachelomonas* spp. | River | exp | 30.97 | 63.95 | 0.000 | 0.533 |
| All taxa | River | norm | 1.23 | 6.45 | 3.094 | 1.034 |
| All taxa | River | lognorm | 0.19 | 4.39 | 1.027 | 0.000 |
| All taxa | River | gamma | 3.05 | 10.10 | 6.742 | 2.858 |
| All taxa | River | chisq | 3.48 | 8.95 | 5.594 | 3.284 |
| All taxa | River | exp | 0.68 | 3.36 | 0.000 | 0.487 |
| Diatoms | River | norm | 1.10 | 6.20 | 2.776 | 0.506 |
| Diatoms | River | lognorm | 0.59 | 5.19 | 1.765 | 0.000 |
| Diatoms | River | gamma | 3.70 | 11.39 | 7.968 | 3.102 |
| Diatoms | River | chisq | 2.80 | 7.61 | 4.183 | 2.209 |
| Diatoms | River | exp | 0.71 | 3.42 | 0.000 | 0.118 |
| Colonial | River | norm | 1.70 | 7.39 | 2.657 | 0.802 |
| Colonial | River | lognorm | 0.89 | 5.79 | 1.054 | 0.000 |
| Colonial | River | gamma | 5.55 | 15.10 | 10.366 | 4.656 |
| Colonial | River | chisq | 3.60 | 9.21 | 4.472 | 2.709 |
| Colonial | River | exp | 1.37 | 4.73 | 0.000 | 0.473 |
| Cyanobacteria | River | norm | 107.79 | 219.58 | 36.378 | 17.189 |
| Cyanobacteria | River | lognorm | 106.27 | 216.54 | 33.334 | 15.667 |
| Cyanobacteria | River | gamma | 126.47 | 256.94 | 73.738 | 35.869 |
| Cyanobacteria | River | chisq | 90.60 | 183.20 | 0.000 | 0.000 |
| Cyanobacteria | River | exp | 92.99 | 187.98 | 4.775 | 2.388 |
| Filamentous | River | norm | 5.53 | 15.05 | 2.503 | 0.977 |
| Filamentous | River | lognorm | 4.55 | 13.10 | 0.549 | 0.000 |
| Filamentous | River | gamma | 9.63 | 23.27 | 10.720 | 5.086 |
| Filamentous | River | chisq | 7.75 | 17.50 | 4.956 | 3.204 |
| Filamentous | River | exp | 5.27 | 12.55 | 0.000 | 0.726 |
| Flagellates | River | norm | 11.01 | 26.02 | 2.454 | 0.592 |
| Flagellates | River | lognorm | 10.42 | 24.84 | 1.270 | 0.000 |
| Flagellates | River | gamma | 19.16 | 42.32 | 18.756 | 8.743 |
| Flagellates | River | chisq | 13.12 | 28.24 | 4.674 | 2.702 |
| Flagellates | River | exp | 10.78 | 23.57 | 0.000 | 0.365 |
| Single cells | River | norm | 0.32 | 4.63 | 1.968 | 0.076 |
| Single cells | River | lognorm | 0.24 | 4.48 | 1.817 | 0.000 |
| Single cells | River | gamma | 0.46 | 4.92 | 2.258 | 0.221 |
| Single cells | River | chisq | 7.00 | 15.99 | 13.325 | 6.754 |
| Single cells | River | exp | 0.33 | 2.67 | 0.000 | 0.092 |

# References

Bowman, A. W., and A. Azzalini. 1997. Applied Smoothing Techniques for Data Analysis: The Kernel Approach with S-Plus Illustrations, OUP Oxford.

Dingman, S.L. 2009. Fluvial Hydraulics. Oxford UP, New York.

Hicks, D. M. 1991. Roughness characteristics of New Zealand rivers: a handbook for assigning hydraulic roughness coefficients to river reaches by the “visual comparison” approach, Water Resources Survey, DSIR Marine and Freshwater.

Jones, M. C. 1993. Simple boundary correction for kernel density estimation. Stat Comput **3**: 135–146. doi:10.1007/BF00147776

Nathan, R., E. K. Klein, J. J. Robledo-Arnuncio, and E. Revilla. 2012. Dispersal kernels, p. 187–210. *In* J. Clobert [ed.], Dispersal ecology and evolution. Oxford University Press Oxford.

Silverman, B. W. 2017. Density Estimation for Statistics and Data Analysis, Routledge.

Wand, M. P., and M. C. Jones. 1994. Kernel Smoothing, CRC Press.
